# Potential‐Mediated Recycling of Copper From Brackish Water by an Electrochemical Copper Pump

**DOI:** 10.1002/advs.202203189

**Published:** 2022-08-26

**Authors:** Hai Deng, Wenfei Wei, Lei Yao, Zijian Zheng, Bei Li, Amr Abdelkader, Libo Deng

**Affiliations:** ^1^ College of Chemistry and Environmental Engineering Shenzhen University Shenzhen 518060 P. R. China; ^2^ Shenzhen Key Laboratory of Special Functional Materials Shenzhen Engineering Laboratory for Advanced Technology of Ceramics Guangdong Research Center for Interfacial Engineering of Functional Materials College of Materials Science and Engineering Shenzhen University Shenzhen 518060 P. R. China; ^3^ Institute of Textiles and Clothing Research Institute for Smart Energy The Hong Kong Polytechnic University Hong Kong SAR P. R. China; ^4^ College of Biology and the Environment Nanjing Forestry University Nanjing 210037 P. R. China; ^5^ Department of Design and Engineering Faculty of Science & Technology Bournemouth University Poole Dorset BH12 5BB UK

**Keywords:** capacitive deionization, electrochemical copper pump, electrodeposition, hierarchical porous carbon nanosheets

## Abstract

Copper ions (Cu^2+^) disposed to the environment at massive scale pose severe threat to human health and waste of resource. Electrochemical deionization (EDI) which captures ions by electrical field is a promising technique for water purification. However, the removal capacity and selectivity toward Cu^2+^ are unsatisfying, yet the recycling of the captured copper in EDI systems is yet to be explored. Herein, an efficient electrochemical copper pump (ECP) that can deliver Cu^2+^ from dilute brackish water into much more concentrated solutions is constructed using carbon nanosheets for the first time, which works based on reversible electrosorption and electrodeposition. The trade‐off between the removal capacity and reversibility is mediated by the operation voltage. The ECP exhibits a removal capacity of 702.5 mg g^−1^ toward Cu^2+^ and a high selectivity coefficient of 64 for Cu^2+^/Na^+^ in the presence of multiple cations; both are the highest reported to date. The energy consumption of 1.79 Wh g^–1^ is among the lowest for EDI of copper. More importantly, the Cu species captured can be released into a 20‐fold higher concentrated solution. Such a high performance is attributed to the optimal potential distribution between the two electrodes that allows reversible electrodeposition and efficient electrosorption.

## Graphical abstract

A novel electrochemical copper pump that can deliver Cu^2+^ from brackish water into a 20‐fold higher concentrated solution is constructed using hierarchical porous carbon nanosheets. The pump exhibits the highest removal capacity and selectivity toward Cu^2+^ so far and can release the captured Cu species efficiently; all are enabled by reversible electrosorption and electrodeposition at high operation voltages.

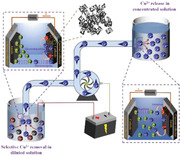

## 1 Introduction

With the rapid growth of mining, plating, and printing circuit industries, massive amounts of wastewater containing copper ion (Cu^2+^) are being discharged into the environment, which poses a threat to human health and the ecological system and causes waste of valuable resources.^[^ ^1^ ^]^ Removal and/or recovery of Cu^2+^ from the wastewater is thus an important topic. Several techniques have been developed for such tasks, including chemical precipitation, ion exchange, membrane filtration, and electrocoagulation.^[^ ^2^ ^]^ However, it is still a great challenge to recycle Cu^2+^ from dilute brackish water in which the concentration of Cu^2+^ is typically below 1000 ppm. The consensus is that techniques with even higher selectivity, lower costs, and less secondary pollution are needed to meet the future demands.

Capacitive deionization (CDI), which removes ions through electrostatic attraction or Faradaic reaction under electric potential, is an emerging electrochemical desalination technology attracting enormous interest recently.^[^ ^3^ ^]^ Theoretically, the captured ions can be repelled from the electrode and released back into the solution so that the electrode is refreshed and the valuable ions can be potentially recovered.^[^ ^4^ ^]^ Compared to the conventional techniques, CDI has a range of advantages such as easy maintenance of the equipment, low secondary pollution, and high efficiency for desalination of brackish water.^[^ ^5^ ^]^

Electrodes are the heart of a CDI system, which are most viably constructed using porous carbons due to their low cost, good electrical conductivity, and chemical stability.^[^ ^6^ ^]^ However, the adsorption capacity of carbonaceous electrodes toward Cu^2+^ is still unsatisfying, for example, the highest adsorption capacity for Cu^2+^ was only 77.8 mg g^–1^ with a cation‐exchange resin‐derived carbon.^[^ ^7^ ^]^ Furthermore, an even more challenging aspect for practical application is to selectively remove Cu^2+^ among the competing cations. The selectivity coefficients for Cu^2+^ over alkaline cations based on porous carbons have been rather low, typically below 5,^[^ ^8^ ^]^ which is restricted intrinsically by the selection mechanisms such as ion sieving, surface, and electrostatic affinity, difference in mobility, hydration energy, hydration ratio, affinity toward functional groups, and electronegativity.^[^ ^9^ ^]^ A selectivity coefficient for Cu^2+^/Na^+^ up to 30 was achieved by introducing pseudocapacitive FeS_2_ into porous carbon but the cyclic stability is deteriorated.^[^ ^10^ ^]^ Therefore, substantial improvement for the removal capacity and selectivity of the current CDI systems is needed. What's more, an equally important aspect and a trade‐off with the capturing capability of the electrode, namely the recycling of the valuable Cu^2+^ captured in the CDI system, has not been explored so far.

On the other hand, electrodeposition is a technology that has been extensively used in the industry for removal/recovery of metals and is still attracting great research interest recently. When dealing with electrodeposition of copper, a wide range of concentrations of wastewater, from tens of ppb to tens of g L^–1^ can be treated using this technology. Furthermore, high‐quality copper can be deposited on various substrates such as on stainless steel, graphite, and carbon cloth. Recent studies have been focused mostly on enhancing the deposition speed, the mass transfer, the energy efficiency, the selectivity, and so on.^[^ ^11^ ^]^ However, the state‐of‐art energy consumption is still as high as 3.5 Wh g^–1^ for the electrodeposition process.^[^ ^12^ ^]^ Moreover, the cyclic stability of the electrode, which is crucial for the equipment maintenance and cost of the process, has not been explored so far.

In this work, we report for the first time, an electrochemical copper pump (ECP) that can capture Cu^2+^ from dilute brackish water and release them into a more concentrated solution, with the ions driven by the electrical field. This system was constructed using hierarchical porous carbon nanosheets, which allows reversible electrosorption and electrodeposition at high voltages. Specifically, a new aromatic polymer poly(phenylenediamine‐co‐phthalaldehyde) (PPDPA), synthesized through condensation between p‐phenylenediamine and 1, 4‐phthalaldehyde, was utilized as the carbon source. The rigid aromatic backbone of PPDPA is readily converted into highly graphitized carbon network with optimal pore structure upon carbonization and KOH activation. The ECP constructed with the optimal electrode material exhibited a removal capacity of 702.5 mg g^−1^ toward Cu^2+^ at an initial concentration of 400 ppm and a voltage of 1.2 V and a high selectivity coefficient of 64 for Cu^2+^/Na^+^ in the presence of multiple cations; both are the highest for carbon electrodes reported to date. The lowest energy consumption was 1.79 Wh g^–1^ Cu at an initial concentration of 50 ppm, which is significantly lower than those treated by other electrochemical systems for copper removal. More importantly, the Cu species captured can be released into a 20‐fold higher concentrated solution. Such an efficient ECP is attributed to the optimal potential distribution between the two electrodes that allows reversible electrodeposition and robust electrosorption.

## 2 Results and Discussion

### 2.1 Synthesis and Characterization

**Figure 1:**
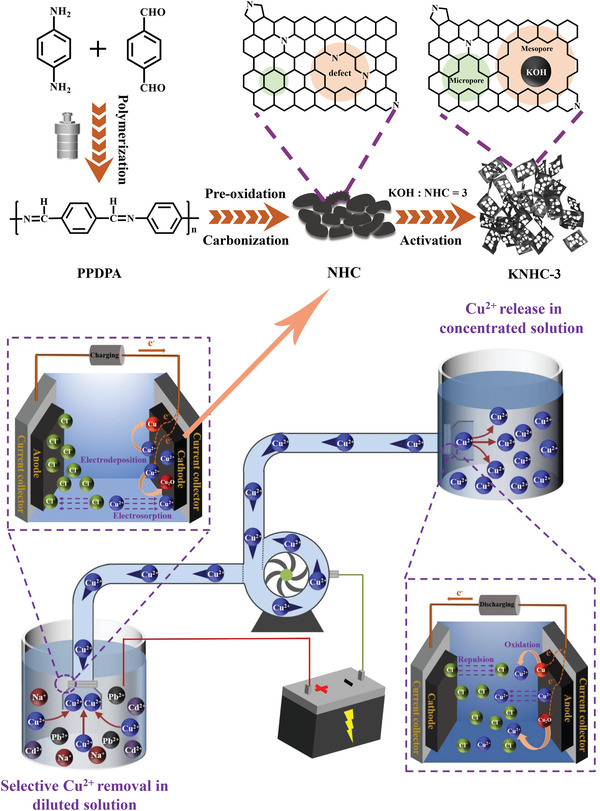
Schematic diagram of the ECP and the preparation procedure of the electrode material (KNHC‐3).

The electrode material that has a high capacity and selectivity toward Cu^2+^ is crucial to construct an ECP that can deliver Cu^2+^ from brackish water into a more concentrated solution (**Figure** **1**). Preparation of such electrode materials was started with synthesis of an aromatic copolymer PPDPA that consists of rigid, aromatic, and sp^2^‐hybridized molecular backbone. The condensation polymerization between p‐phenylenediamine and 1, 4‐phthalaldehyde was confirmed by ^13^C‐nuclear magnetic resonance (NMR) spectrum of the as‐prepared polymer (Figure S1a, Supporting Information). The cluster of peaks located in the range of 110 to 155 ppm and 160 ppm corresponds to the aromatic carbons and the C=N carbon,^[^ ^13^ ^]^ respectively. This was further corroborated with the Fourier transform infrared (FTIR) spectrum (Figure S1b, Supporting Information), which showed the stretching vibration at 1610 cm^–1^ corresponding to the C=N bond formed by the reaction between the amine and aldehyde groups.^[^ ^14^ ^]^ The thermogravimetric analysis (TGA) curve indicates that this aromatic polymer has excellent thermal stability, that no significant degradation occurs until 500 °C, and a high carbon yield (34%) could be obtained at 900 °C (Figure S1c, Supporting Information). The reason for using PPDPA as the precursor is that carbonization of this aromatic polymer could result in graphitic layers incorporated in amorphous and defective carbons, and KOH etching would occur preferentially in the less stable regions as demonstrated in our previous studies,^[^ ^15^ ^]^ creating hierarchical pores. This could potentially balance the interplay among the surface area, porosity, surface functional group, and graphitization degree in a carbon material, and thus, optimize the electrochemical performance.^[^ ^15a^ ^]^ The morphology, composition, and pore structure of the PPDPA‐derived carbons were systematically investigated. Upon KOH activation, the pyrolytic carbon evolved from a dense and laminated structure to small flakes (the products prepared with different mass ratio between KOH and PPDPA‐derived carbon were denoted as KNHC‐*n* where *n* = 1, 2, and 3, and the scanning electron microscope (SEM) images are shown in Figure S2, Supporting Information), and finally into carbon nanosheets which is the intrinsic morphology of graphitic domain (Figure S3a, Supporting Information).

**Figure 2:**
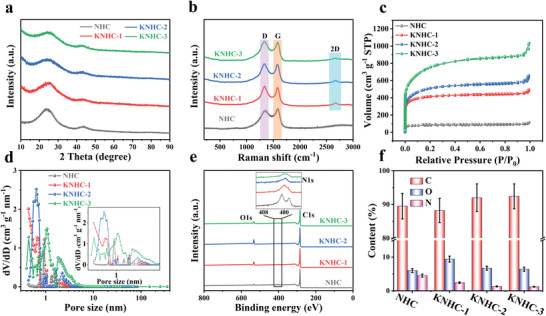
a) XRD patterns, b) Raman spectra, c) N_2_ adsorption–desorption isotherms, d) pore size distribution plots, e) XPS full survey spectra, and f) the element contents of NHC, KNHC‐1, KNHC‐2, and KNHC‐3, in which the error bars represent the standard deviation (sample size: 3).

X‐ray diffraction (XRD) patterns in **Figure** **2**a revealed predominantly amorphous nature with polycrystalline domain (i.e., turbostratic structure), evident by the broad reflections at 25° and 43° corresponding to the (002) and (101) planes,^[^ ^16^ ^]^ respectively. The sp^2^‐hybridized carbon with substantial disorder was also revealed by the presence of G‐ and D‐bands at 1586 cm^−1^ and 1340 cm^−1^ in the Raman spectra (Figure 2b).^[^ ^17^ ^]^ Furthermore, a 2D‐band at 2648 cm^−1^ was seen for all the activated carbons. This indicated that the graphitic domains, while beneficial for the electrochemical performance, were formed at a relatively low temperature due to the conjugate structure of PPDPA and the KOH templating effect.^[^ ^18^ ^]^ The N_2_ adsorption–desorption isotherms for the activated carbons display major adsorption at low pressure (*P*/*P*_0_ < 0.01) and a crescent‐like hysteresis loop at *P*/*P*_0_ > 0.45 (Figure 2c), indicating the presence of hierarchical pores,^[^ ^19^ ^]^ which is better illustrated by the pore size distribution curves in Figure 2d. The specific surface area (SSA) increased from 541 m^2^ g^–1^ for NHC to 2786 m^2^ g^–1^ for KNHC‐3 upon KOH etching of the less stable region, accompanied by the increase of the average pore size from 1.25 to 2.20 nm (Parameters for the pore structure are listed in Table S1, Supporting Information). Further increasing the amount of KOH resulted in a rather low carbon yield (2%) and is not sensible for practical application. The full X‐ray photoelectron spectroscopy (XPS) spectra in Figure 2e manifested the successful doping of both O and N elements in all samples (C 1s, O 1s, and N 1s spectra are shown in Figure S4, Supporting Information). Particularly, the contents of nitrogen and oxygen were 1.2 and 6.4 at% in KNHC‐3, respectively (Figure 2f).

### 2.2 Electrochemical Properties

**Figure 3:**
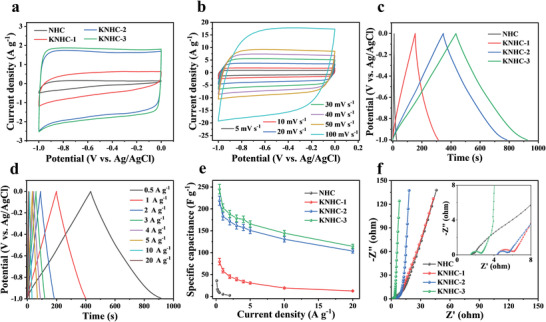
Electrochemical tests in a three‐electrode system with a 1 m Na_2_SO_4_ electrolyte: a) CV curves of the as‐prepared samples at 10 mV s^–1^, b) CV curves of KNHC‐3 at different scan rates, c) GCD curves at 0.5 A g^–1^, d) GCD curves of KNHC‐3 tested at different current density, e) The specific capacitance as a function of current density, and f) Nyquist plots. The error bars represent the standard deviation (sample size: 3).

The electrochemical properties of the as‐prepared carbons were first evaluated in a three‐electrode system in a concentrated electrolyte (1 m Na_2_SO_4_). The cyclic voltammetry (CV) curve acquired at 10 mV s^–1^ for NHC electrode showed a low current response at low potential (and thus a triangular shape) due to its low SSA and poor porosity,^[^ ^20^ ^]^ which requires a high potential to capture ions (**Figure** **3**a). In contrast, all the curves of activated carbons exhibited a rectangular shape without obvious redox peaks or distortion in the scan rate range of 5 to 100 mV s^–1^ (Figure 3b; Figure S5a–c, Supporting Information), and the corresponding galvanostatic charge–discharge (GCD) curves displayed an isosceles triangular shape without appreciable iR drop (Figure 3c,d; Figure S5d–f, Supporting Information), suggesting efficient ion transport, good electrical conductivity, and high Coulombic efficiencies in these electrodes, which are typical of electrical double layer capacitive behavior.^[^ ^21^ ^]^ Figure 3e shows the specific capacitance (*C*_s_) determined by GCD for all samples. KNHC‐3 showed the highest capacitance at all current densities. For example, at 0.5 A g^–1^, the *C*_s_ of KNHC‐3 electrode was 245 F g^–1^, higher than NHC (9 F g^–1^), KNHC‐1 (80 F g^–1^), and KNHC‐2 (219 F g^–1^). When the current density increased to 20 A g^–1^, the capacitance of KNHC‐3 still retained 46.9% of its value at 0.5 A g^–1^, further indicating the good conductivity and accessibility of the pores in this electrode. The efficient electron and ion transportation kinetics of the carbon electrodes were also reflected by the Nyquist plots (Figure 3f). All the electrodes displayed a semicircular shape in the high‐frequency region and a straight line with a gradual increase of the slope at the low frequency region. The highest slope in low frequency and lowest *R*_ct_ (1.2 Ω) for KNHC‐3 reflects a relatively higher diffusion efficiency, which is consistent with its highest fraction of mesopore shown in Table S1, Supporting Information.^[^ ^22^ ^]^ In all, the high capacitance and good rate performance of KNHC‐3 is thought to be related to the high surface area, optimal pore structure, heteroatom doping, and good conductivity enabled by the high degree of graphitization.

### 2.3 Electrochemical Removal of Cu^2+^

**Figure 4:**
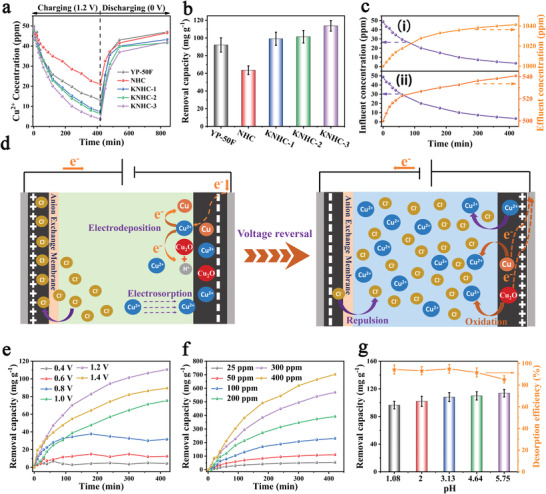
Variation of the Cu^2+^ concentration during charging in a 50 ppm Cu^2+^ solution and discharging under different conditions: a) discharging in deionized solution at 0 V; b) the removal capacity for all carbons; c) charging (purple)–discharging (orange) in more concentrated solutions at −1.2 V for the KNHC‐3 electrode: i) 1000 ppm (20‐fold) and ii) 500 ppm (tenfold); d) schematic diagram of the electrochemical system for Cu^2+^ release in concentrated solutions; e–g) Cu^2+^ removal capacity of KNHC‐3 electrode under different voltages, initial concentrations, and pH values. The error bars represent the standard deviation (sample size: 3).

The above results suggested that KHNC‐3 has excellent capacitive performance in concentrated electrolytes, and thus, its application for electrochemical removal of ions in brackish water was further tested using a batch‐mode electrochemical deionization system in a 50 ppm Cu^2+^ solution. The system was charged at a constant voltage of 1.2 V for 420 min and then discharged at 0 V. As can be seen from **Figure** **4**a, the Cu^2+^ concentration decreased during charging and recovered almost its initial value upon discharging at 0 V. Among the four PPDPA‐derived carbons, the KNHC‐3 electrode exhibited the highest Cu^2+^ removal capacity, of 113.7 mg g^–1^ (corresponding to a removal percentage of 92.8%, that is, from 50 to 3.6 ppm, Figure 4b). As a comparison, a typical commercial activated carbon (YP‐50F) exhibited a removal capacity of 92.1 mg g^–1^ at the same testing condition, which is 19.0% lower than the KNHC‐3. The higher capacity of KNHC‐3 than other carbons was also reflected by the corresponding current response shown in Figure S6a, Supporting Information, in which KNHC‐3 showed the highest current. Moreover, the Ragone plots of KNHC‐3 shifted toward the upper right region, indicating a simultaneously faster removal rate and higher removal capacity (Figure S6b, Supporting Information).

After simply discharging at 0 V, the Cu^2+^ concentration in the effluent increased from 3.6 to 43.1 ppm, indicating that 85.1% of the Cu species captured by the electrode during charging was released into the deionized solution. Most strikingly, the Cu species captured could also be efficiently released into a significantly higher concentrated solution by reversing the voltage (in this case, an anion exchange membrane was attached to the anode), showing a concentrating function of the pump. For example, 93.9% and 91.3% of the Cu species captured in the 50 ppm Cu^2+^ solution could be released into 10‐fold (500 ppm) and 20‐fold (1000 ppm) more concentrated solutions (Figure 4c; Figure S7a, Supporting Information), that is, the ECP displayed a concentration ratio (defined as the ratio of the concentrations of the highest discharge solution and the feed solution) up to 20. Moreover, the residual Cu species on the electrode after discharging were negligible (i.e., 0.21 and 0.35 at% after discharging in 500 and 1000 ppm Cu^2+^ solutions, respectively, Figure S7b, Supporting Information), thus acting as a robust ECP that can efficiently deliver Cu^2+^ from dilute brackish water to concentrated solutions (Figure 4d). Such a pump relies critically on the high removal capacity of the KNHC‐3 electrode (which is far superior to the leading results tested at similar conditions for carbon electrodes reported so far, Table S2, Supporting Information), as well as the efficient release of the captured Cu species.^[^ ^7^ ^]^

The effects of testing condition including the voltage, initial concentration, and pH on the removal performance of the ECP were analyzed in detail. Deionization curves acquired in a 50 ppm solution at different voltages are shown in Figure 4e. As expected, the Cu^2+^ removal capacity increased with the voltage. Particularly, the removal capacity increased abruptly when the voltage exceeded 0.6 V and reached its maximum at 1.2 V (it decreased at 1.4 V due to parasitic reactions). The increase of removal capacity was accompanied with a higher energy efficiency at higher voltages. As can be seen from Figure S8, Supporting Information, the energy consumption of the KNHC‐3‐based ECP decreased from 5.45 Wh g^–1^ Cu at 0.6 V to 1.79 Wh g^–1^ Cu at 1.2 V when operated in a 50 ppm solution. The value is significantly lower than those reported for other electrochemical systems for removal of copper, such as an energy consumption of 2.0 Wh g^–1^ for the electrosorption–electrodeposition combined process,^[^ ^8a^ ^]^ and 3.5 Wh g^–1^ for the sole electrodeposition.^[^ ^12^ ^]^ Moreover, the Ragone plots at 1.2 V shifted toward the upper right region relative to the other voltages, indicating a simultaneously faster removal rate and higher removal capacity (Figure S9, Supporting Information). The deionization kinetic curve can be fitted by both pseudo‐first‐order and pseudo‐second‐order models (Figure S10 and Table S3, Supporting Information), indicating that both physical adsorption and chemical adsorption are the rate‐limiting steps during the Cu^2+^ removal process.^[^ ^23^ ^]^

The removal capacity also increased with the initial concentration (Figure 4f), from 53.5 mg g^–1^ at 25 ppm to 702.5 mg g^–1^ at 400 ppm. The influence of pH value on the removal capacity is shown in Figure 4g. The Cu^2+^ removal capacity decreased monotonically with the decrease of solution pH, from 113.7 mg g^–1^ at pH 5.75 to 96.5 mg g^–1^ at pH 1.08, indicating that the acidic environment has a negative effect on Cu^2+^ removal due to the competition between Cu^2+^ and H^+^.^[^ ^24^ ^]^ However, the desorption efficiency gradually increased with the decrease of pH, indicating that a more acidic solution is beneficial to the release of Cu^2+^, which was consistent with the studies reported by Gui et al. with a graphite sheet.^[^ ^25^ ^]^

The cycling stability of the ECP is also crucial for practical application, which was tested in a 50 ppm solution at pH 5.75 and 1.2 V. As can be seen from Figure S11, Supporting Information, KNHC‐3 still exhibited a high removal capacity of 94.2 mg g^–1^ after five charge–discharge cycles, that is, 85.3% of the initial capacity was retained. An even more challenging aspect is the regeneration of electrode in a higher‐concentrated solution (1000 ppm). As can be seen from Figure S12, Supporting Information, the desorption efficiency (i.e., the ratio between the released species and the captured species) only decreased slightly from 91.3% to 85.6% after five capture–release cycles. The high desorption efficiency was further confirmed by XRD and XPS characterizations, where no peaks for Cu species were observed in KNHC‐3 electrode using these techniques after five capture–release cycles (Figure S13, Supporting Information). These results indicated that KNHC‐3 electrode has excellent cycling stability and reversibility. It should be noted the total charging duration was 35 h within five cycles in this case, which is longer than most of the reported operation durations in literature, such as 3 h for 3D rGO and 10 h for activated carbon.^[^ ^24^, ^26^ ^]^

### 2.4 Mechanism for Electrochemical Removal of Cu^2+^

The mechanism for the removal of Cu^2+^ was further explored. Cu^2+^ ions were first adsorbed on the cathode due to electrostatic interaction, which might be reduced to metal copper (Cu) and cuprous oxide (Cu_2_O) through the following reactions at different potentials:^[^ ^27^ ^]^

(1) $${\mathrm{Cu}}^{2+}+2e^{-}\to \;\mathrm{Cu}\;\left(E^{0}=0.340\;V\;\mathrm{vs}\;\mathrm{SHE}\right)$$

(2) $$2{\mathrm{Cu}}^{2+}+H_{2}O+2e^{-}\to {\mathrm{Cu}}_{2}O+2H^{+}\left(E^{0}=0.203\;V\;\mathrm{vs}\;\mathrm{SHE}\;\right)$$

where *E* ^0^ is the standard electrode potential. During charging, the equilibrium electrode potential (*E*_w_) would change due to the continuous variations of pH and Cu^2+^ concentration (Figure S14, Supporting Information) and could be calculated based on the Nernst equation given by Equations (3) and (4):

(3) $$E_{w}\left({\mathrm{Cu}}^{2+}/\mathrm{Cu}\right)=E^{0}\left({\mathrm{Cu}}^{2+}/\mathrm{Cu}\right)-\frac{\mathrm{RT}}{nF}ln\frac{1}{\left[{\mathrm{Cu}}^{2+}\right]}$$

(4) $$E_{w}\left({\mathrm{Cu}}^{2+}/{\mathrm{Cu}}_{2}O\right)=E^{0}\left({\mathrm{Cu}}^{2+}/{\mathrm{Cu}}_{2}O\right)-\frac{\mathrm{RT}}{nF}ln\frac{{\left[H^{+}\right]}^{2}}{{\left[{\mathrm{Cu}}^{2+}\right]}^{2}}$$

where F is the Faraday constant (9.6485 × 10^4^ C mol^–1^), R is the gas constant (8.314 J K^–1^ mol^–1^), *T* is the absolute temperature (298 K), and *n* is the number of moles of electrons transferred in the half‐reactions.^[^ ^28^ ^]^

**Figure 5:**
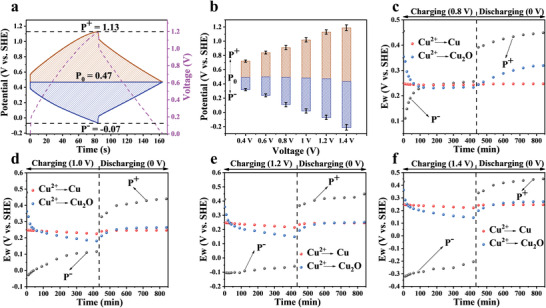
a) GCD curves of the anode and cathode in 50 ppm Cu^2+^ solution with a supporting electrolyte of Na_2_SO_4_ at voltage of 1.2 V, and the current density was 0.5 A g^–1^, b) potential distribution of KNHC‐3 electrode at different voltages; *E_w_*, P^–^ and P^+^ during the deionization tests operated at: c) 0.8 V, d) 1 V, e) 1.2 V, and f) 1.4 V. The error bars represent the standard deviation (sample size: 3).

The development of potential between the two electrodes in the pump was first assessed with an additional reference electrode (see details in the Experimental section; Figure S15a, Supporting Information). As can be seen from **Figure** **5**a, when the voltage was set at 1.2 V, the anode potential (P^+^) and cathode potential (P^–^) reached 1.13 V and –0.07 V, respectively, with a potential at zero voltage (P_0_) of 0.47 V (vs SHE), all falling into the safe window that avoids the electrolysis of the electrolyte. The cathode potential decreased monotonically with the increase of voltage applied to the pump (Figure 5b; Figure S15b–f, Supporting Information). The dynamic potential distribution was further monitored during charging and discharging. Reduction reactions would occur when the *E*_w_ was higher than P^–^ whereas oxidation reactions would occur when the *E*_w_ was lower than P^+^.^[^ ^29^ ^]^ As shown in Figure S16a, Supporting Information, *E*_w_ of both Reactions (1) and (2) were lower than P^–^ when charged at 0.4 V, meaning that only electrosorption occurred. Notably, although *E*_w_(Cu^2+^/Cu_2_O) was already above P^–^ in the early stage when charged at 0.6 V (Figure S16b, Supporting Information), the reduction of Cu^2+^ was negligible due to the presence of overpotential. When the voltage exceeded 0.8 V, Cu^2+^ could be reduced to Cu_2_O and Cu as both *E*_w_ (Cu^2+^/Cu_2_O) and *E*_w_ (Cu^2+^/Cu) were above P^–^ (Figure 5c–f), in which the reduction of Cu^2+^ to Cu_2_O is more favorable than to Cu at 0.8 V due to a higher value of *E*_w_ (Cu^2+^/Cu_2_O). Interestingly, the value of *E*_w_ (Cu^2+^/Cu_2_O) became lower than *E*_w_ (Cu^2+^/Cu) after charging at 1, 1.2, or 1.4 V for a period of time, indicating that the reduction of Cu^2+^ into Cu would become more favorable. These redox reactions can be clearly seen from the CV curves shown in Figure S17, Supporting Information, that is, the peaks at –0.5 and –0.83 V correspond to Cu^2+^/Cu_2_O and Cu^2+^/Cu, respectively. Moreover, the *E*_w_ of these two reactions were both below P^+^ during the discharging process, indicating that the generated Cu_2_O and Cu during charging would be oxidized to Cu^2+^ and released back into the solution. It is worth noting that the variation of P^+^ when operated at –1.2 V in concentrated (500 and 1000 ppm) solutions are similar to that in the deionized and diluted solution (Figure S18, Supporting Information), and thus, oxidation and release of the deposited Cu species into the concentrated solution is expected. In all, the high removal capacity of the ECP is associated with the combination of electrosorption and electrodeposition, and the release of the captured Cu is related to the oxidation of the Cu species. The high electrosorption capacity of KNHC‐3 is attributed to the high surface area and high degree of graphitization, whereas the reversible electrodeposition is achieved through judicious optimization of the operation condition. It is the electrode material and operation voltage that endow the ECP with high capacity, excellent durability, and reversibility.

**Figure 6:**
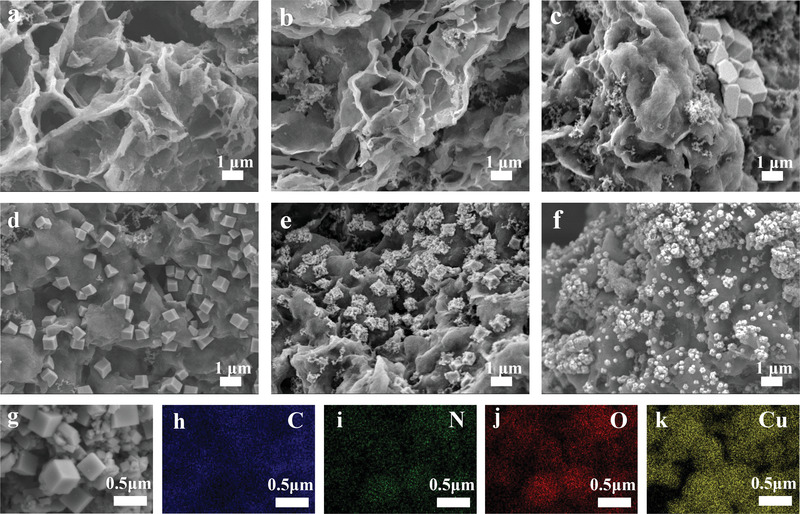
SEM images of the cathode after charging at: a) 0.4 V, b) 0.6 V, c) 0.8 V, d) 1 V, e) 1.2 V, and f) 1.4 V; g–k) EDS mapping for the region of emerging particles after charging at 1.2 V.

The morphology and composition of KNHC‐3 electrode after charging and discharging were analyzed to better understand the removal mechanism. SEM images of the cathode after charging at different voltages are shown in **Figure** **6**. The electrode surface remained clear and smooth without obvious change when charged at 0.4 and 0.6 V (Figure 6a,b), indicating that only electrosorption occurs during this process. In contrast, sparse particles with sharp facets were observed after charging at 0.8 V, suggesting the electrodeposition of Cu^2+^ (Figure 6c). With the increase of voltage, the quantity of particles increased progressively (whereas the size decreased, Figure 6d–f). EDS mapping for the region of emerging particles is shown in Figure 6g–k, which clearly reveals a significant amount of Cu species. These results suggest that the extraordinary removal of Cu^2+^ at 1.2 V is associated with the synergistic effect of electrosorption and electrodeposition, in which both processes are reversible upon simply discharging at 0 V.

**Figure 7:**
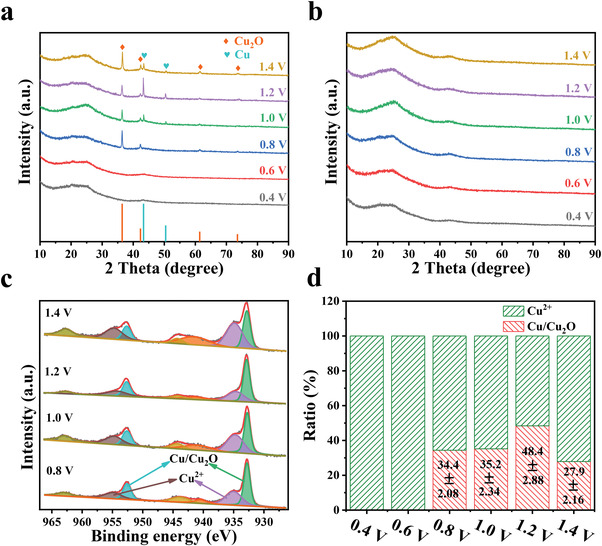
XRD patterns of cathodes operated at different voltages: a) after charging and b) after discharging; c) the high resolution spectrum of Cu 2p and d) the fraction of reduced Cu among the total Cu species on the cathode after charging at different voltages. Data are presented as the mean values ± standard deviation.

The aforementioned mechanism was further verified by XRD characterization of the electrode after charging. As shown in **Figure** **7**a, only diffraction peaks at 2*θ* = 25° and 43° typical of carbon were observed when charged at 0.4 and 0.6 V. When the voltage was increased to 0.8 V, diffraction peaks at 2*θ* = 36.4°, 42.3°, 61.4°, and 73.6° for Cu_2_O (PDF#78‐2076) could be observed. Further increasing the voltage to 1.0 V or above, additional peaks at 2*θ* = 43.3° and 50.5° for Cu (PDF#85‐1326) could be observed, indicating that Cu^2+^ species were reduced to both Cu_2_O and Cu at these voltages. In addition, the XRD patterns for the cathode after discharging (Figure 7b) and the anode after both charging and discharging (Figure S19, Supporting Information) all displayed only two diffraction peaks for carbon but without Cu or Cu_2_O diffraction peaks at any of these operation voltages, which confirms the excellent reversibility of the KNHC‐3 electrode.

The content and valence states of Cu element on the surface of cathode after charging and discharging were analyzed by XPS. The high‐resolution spectrum of Cu 2p after charging at 0.4 and 0.6 V all displayed two peaks at binding energies of 933.9 and 953.7 eV (Figure S20, Supporting Information), confirming that Cu element on KNHC‐3 surface was Cu^2+^.^[^ ^30^ ^]^ When the voltage was increased to 0.8–1.4 V (Figure 7c), in addition to the Cu 2p_3/2_ peak at 934.6–934.9 eV and the Cu 2p_1/2_ peak at 954.7–954.8 eV attributed to Cu^2+^,^[^ ^31^ ^]^ another two distinct peaks at 932.6–932.9 and 952.5–952.7 eV corresponding to Cu/Cu_2_O were also observed.^[^ ^32^ ^]^ More specifically, the reduced forms of Cu^2+^ (i.e., Cu and Cu_2_O) account for 0%, 0%, 34.4%, 35.2%, 48.4%, and 27.9% at 0.4, 0.6, 0.8, 1.0, 1.2, and 1.4 V, respectively (Figure 7d). However, it is difficult to distinguish Cu from Cu_2_O in the Cu 2p spectrum due to similar binding energies for these two species. As such, LMM Auger spectra of copper were further acquired. As shown in Figure S21, Supporting Information, the peak centered at 569.4 eV is assigned to Cu_2_O, and three other peaks at 572.4, 566.7, and 565.6 eV represent different transition states of the Cu LMM spectrum.^[^ ^33^ ^]^

The atomic ratio of Cu element on the cathode surface after charging was increased from 0.49% (0.4 V) to 10.2% (1.2 V), and decreased to 8.9% at 1.4 V (Figure S22a, Supporting Information), which was consistent with the variation of Cu^2+^ removal capacity as a function of the voltage. More importantly, the atomic ratio of Cu element for the cathode after discharging and the anode after charging and discharging were all at a low level, ≈0.4% (Figure S22b, Supporting Information), again verifying the excellent regeneration of KNHC‐3 electrode which is crucial for the ECP.

### 2.5 Selective Removal of Cu^2+^

**Figure 8:**
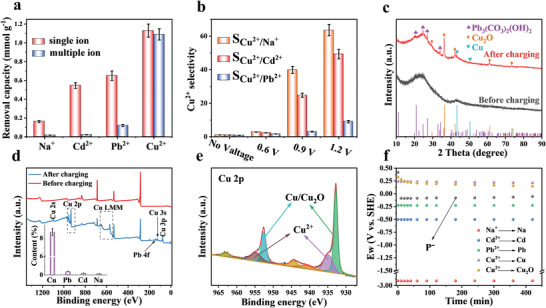
a) Cation removal capacity in individual and mixed solutions under the voltage of 1.2 V, b) Cu^2+^ selectivity coefficient in mixed solution under different voltage, c) XRD patterns, d) XPS full spectra, and e) Cu 2p XPS spectrum of the cathode in the mixed solution after charging at 1.2 V; f) *E_w_* and P^−^ during charging process in the mixed solution operated at 1.2 V. The error bars represent the standard deviation (sample size: 3).

Selective removal of a specific ion in the presence of competing ions is vital as not all ions need to be removed in wastewater. The performance of KNHC‐3 for electrochemical removal of different cations was first evaluated in a mixed solution containing multiple chlorides, including CuCl_2_, CdCl_2_, PbCl_2_, and NaCl, with the initial concentration of all metal ions set at 0.5 mmol L^–1^. For comparison, the removal capacities for all these cations were also measured in each individual solution. **Figure** **8**a compares the removal capacity for each cation measured at 1.2 V with and without competing cations. The removal capacity of Cu^2+^ was 1.09 mmol g^–1^ in the mixed solution, which was much higher than Pb^2+^ (0.121 mmol g^–1^), Cd^2+^ (0.022 mmol g^–1^), and Na^+^ (0.017 mmol g^–1^), and the order of removal capacity in the mixed solution was in good agreement with that measured in each individual solution. Notably, the removal capacity for Cu^2+^ in the mixed solution was only slightly lower than that measured in an individual CuCl_2_ solution. In contrast, the removal capacity of other competing metal ions in the mixed solution was much lower than those in each individual solution, indicating that the system has a superior selectivity toward Cu^2+^ over Pb^2+^, Cd^2+^, and Na^+^.

In fact, there is no preference for Cu^2+^ over other competing cations when a voltage below 0.6 V is applied (Figure 8b). The Cu^2+^ selectivity coefficient sharply increased when the voltage was increased to 0.9 V and reached the maximum value at 1.2 V, namely 64, 49, and 9 versus Na^+^, Cd^2+^, and Pb^2+^, respectively. A closer inspection into the deionization process revealed that the concentration of Na^+^ decreased with a faster rate than Cu^2+^ in the first 30 min during charging due to a higher mobility of Na^+^ (Figure S23, Supporting Information). Afterward, the concentration of Na^+^ gradually increased, indicating that the previously adsorbed Na^+^ was replaced by Cu^2+^ due to a higher charge density (and thus, higher electrostatic attraction) of the latter.^[^ ^34^ ^]^

XRD and XPS analysis were again conducted to investigate the mechanism for selective removal of Cu^2+^ by the KNHC‐3 electrode. XRD patterns of the cathode after charging at 1.2 V in the mixed solution are shown in Figure 8c. Compared to the virgin electrode, diffraction peaks for Cu and Cu_2_O could be observed after charging, suggesting that Cu^2+^ electrodeposition occurred during this process. Notably, diffraction peaks for other precipitants, hydrocerussite (Pb_3_(CO_3_)_2_(OH)_2_), were also observed due to the reaction of Pb^2+^ with the dissolved CO_2_,^[^ ^25^ ^]^ but the valence of Pb^2+^ remained unchanged. Therefore, only Cu^2+^ was reduced among the four cations in the mixed solution, similar to that operated without the competing cations (Figure S24, Supporting Information). These results were also corroborated with XPS analysis. Full XPS spectra of the cathode are shown in Figure 8d, from which additional Cu and Pb elements can be observed after charging, and the atomic contents were 8.68% and 0.37%, respectively (inset in Figure 8d). The high resolution Cu 2p XPS spectrum was deconvoluted and is presented in Figure 8e. The simultaneous appearance of Cu^2+^, Cu_2_O, and Cu peaks indicated that cathodic electrodeposition occurred. In contrast, high resolution Pb 4f XPS spectrum (Figure S25, Supporting Information) revealed that all the Pb species on KNHC‐3 electrode were bivalent,^[^ ^35^ ^]^ which was in good agreement with that operated in the individual PbCl_2_ solution (Figure S26, Supporting Information).

In order to further investigate the mechanism for selective removal of Cu^2+^ in the mixed solution, the dynamic potential distribution during charging at 1.2 V was also measured, and the corresponding *E*_w_ was calculated according to the real time ion concentration and pH (Figure S23, Supporting Information). As shown in Figure 8f, *E*_w_ (Cu^2+^/Cu_2_O) and *E*_w_ (Cu^2+^/Cu) were all above P^–^, implying that Cu^2+^ could be reduced to Cu_2_O and Cu. In contrast, *E*_w_ (Pb^2+^/Pb), *E*_w_ (Cd^2+^/Cd), and *E*_w_ (Na^+^/Na) were all below P^–^, and thus, no reduction was expected for these cations. These results suggest that selective removal of Cu^2+^ over Na^+^ is related to a combination of enhanced electrosorption and cathodic electrodeposition. However, its preference over the other bivalent cations is thought to be associated mainly with electrodeposition, which indicates that 48.4% of the total Cu species exist as the reduced form, and thus, the amount of Cu^2+^ adsorbed (1.13 × [100–48.4%] = 0.578 mmol) is similar to that of the Cd^2+^ adsorbed (0.546 mmol). Selective removal/recovery of cations by carbonaceous electrodes has been previously realized mainly through ion sieving, surface and electrostatic affinity, difference in mobility, hydration energy, hydration ratio, affinity toward functional groups, and electronegativity.^[^ ^9^ ^]^ These mechanisms usually give rise to a selectivity coefficient typically below 5.^[^ ^36^ ^]^ It should be noted that the selective removal of Cu^2+^ is not unique to KHNC‐3. All the PPDPA‐derived carbons and the commercial YP‐50F displayed preference to Cu (Figures S27 and S28, Supporting Information), with the selectivity coefficient increasing with the operation voltage (the Cu^2+^ removal performance of YP‐50F in individual and mixed solution is rather inferior to KNHC‐3 though; see Figure S29, Supporting Information). The tremendous selectivity coefficient obtained here suggests that the selectivity can be enhanced by electrodeposition, provided that the electrode material allows a fast and reversible redox of the target ions at a suitable voltage such as the KNHC‐3 shown here.

## 3 Conclusion

In this study, an efficient ECP was constructed using a hierarchical porous carbon prepared through carbonization and KOH activation of an aromatic polymer. The rigid and aromatic backbone of the precursor allowed facile conversion into graphitic domain with good stability and conductivity, as well as favorable pore structure. The ECP based on the best sample KNHC‐3 showed a Cu^2+^ removal capacity of 702.5 mg g^–1^ in a 400 ppm solution at 1.2 V, which is the highest reported so far. It also showed the lowest energy consumption, of 1.79 Wh g^–1^ Cu in a 50 ppm solution. Furthermore, the pump showed preference toward Cu^2+^ over Pb^2+^, Cd^2+^, and Na^+^, with an extraordinary selectivity coefficient of 64 achieved. More importantly, the captured Cu species could be efficiently released to a 20‐fold more concentrated solution, and the capture‐release cycles could be repeated for five times without significant decay. It was demonstrated that the excellent removal capacity and the preference toward Cu^2+^ were attributed to the combination of electrosorption and electrodeposition. The strategy of combining the materials design and the operation condition to enable the reversible capture of valuable elements from diluted solution would pave the way for industrial application of the EDI technology.

## 4 Experimental Section

### Chemicals

P‐phenylenediamine (97%) and 1‐methyl‐2‐pyrrolidinone (NMP, 99%) were purchased from Aladdin Co. Ltd. 1,4‐phthalaldehyde (98%) was purchased from Energy Chemical. Potassium hydroxide (KOH, 95%) and polyvinylidene difluoride (PVDF) were purchased from Macklin Corporation. Ethanol (AR) was purchased from Guanghua Chemicals. Copper chloride (CuCl_2_, 99%), cadmium chloride (CdCl_2_, 99.999%), lead chloride (PbCl_2_, 99.999%), sodium chloride (NaCl, 99.5%), and sodium sulfate (Na_2_SO_4_, 99.5%) were purchased from Sigma–Aldrich. Hydrochloric acid (HCl) and nitric acid (HNO_3_) were purchased from China National Pharmaceutical Group Corporation. The activated carbon YP‐50F was purchased from Kurary Co. Ltd. Japan. All chemicals were used as received without further purification.

### Synthesis of PPDPA

PPDPA was synthesized according to a previous report with slight modification.^[^ ^37^ ^]^ In a typical experiment, p‐phenylenediamine (0.55 g, 5 mmol) was dissolved in 30 mL of ethanol, followed by addition of 1,4‐phthalaldehyde (0.67 g, 5 mmol) and stirred for 60 min. The mixture was transferred into a 100 mL Teflon liner and treated at 150 °C for 15 h to allow the hydrothermal polymerization. The resultant precipitant was collected by filtration and washed with ethanol for three times to remove the un‐polymerized small molecules. After drying at 70 °C, the PPDPA was obtained.

### Preparation of Porous Carbons

To prepare porous carbons, the as‐prepared PPDPA was pre‐oxidized at 250 °C in air flow at a rate of 1 °C min^–1^ for 2 h. It was then transferred into a tubular furnace and carbonized at 900 °C for 2 h under flowing nitrogen with a heating rate of 5 °C min^–1^ and the obtained nitrogen‐doped hierarchical carbon was denoted as NHC. Afterward, the NHC was mixed with KOH with a mass ratio (*n*) of 1:1, 1: 2, and 1:3 and treated at 900 °C for 2 h in a tubular furnace under flowing nitrogen. The obtained product was soaked in 1 m HCl under vigorous stirring for 12 h and then repeatedly washed with deionized water until neutral, which was then dried at 70 °C for 12 h. The obtained KOH‐activated hierarchical porous carbon was denoted as KNHC‐*n* (*n* = 1, 2, 3).

### Structural Characterization

SEM was performed on a JSM‐7800F and TEAM Octane Plus and TEM was conducted on a JEM‐2100 and X‐Max80. Raman spectroscopy was analyzed on a Renishaw INVIA REFLEX spectrometer coupled with a 532 nm laser. XRD patterns were collected using a Bruker/D8 Advance diffractometer (Cu K*α* radiation). Nitrogen sorption isotherms were obtained using a BELSORP/max at 77 K. The specific surface area (SSA) was determined by the Brunauer–Emmett–Teller method and the pore size distributions were analyzed by a non‐linear density functional model. Chemical compositions were determined by XPS which was conducted on an Ultra DLD using a monochromic Al X‐ray source. FTIR spectra were acquired on a Thermo Nicolet Nexus 870 FTIR spectrometer. Solid‐state NMR spectra were recorded on a VNMRS 400 MHz spectrometer. The ion concentrations were measured by ICP‐OES (Avio 200, PE Instruments). TGA was performed using a TG‐DSC (Mettler Instruments).

### Electrochemical Measurements

To prepare a working electrode, slurry was first prepared by mixing the as‐prepared carbons (NHC, KNHC‐*n*), carbon black and PVDF with a mass ratio of 8:1:1 in NMP. The mixed slurry was coated onto a titanium mesh with a diameter of 1.4 cm, and the mass of active material was ≈2 mg, followed by drying at 60 °C for 24 h. CV, GCD, and electrochemical impedance spectroscopy were tested in 1.0 m Na_2_SO_4_ electrolyte on a CHI 760E electrochemical working station, using a three‐electrode system including a working electrode, Pt foil counter electrode, and Ag/AgCl reference electrode (0.2046 V vs SHE). The specific capacitance values (*C*_s_, F g^−1^) were measured from the GCD curves according to Equation (5):

(5) $$C_{S}=\frac{I\;\times \;\Delta t}{m\;\times \;\Delta V}$$

where *m* (g) is the mass of active material, *I* (A) is the current, Δ*t* (s) is the discharge time, and Δ*V* (V) is the potential range.

### Electrochemical Deionization Tests

Electrochemical removal of Cu^2+^ was conducted using a home‐made electrochemical deionization system, which consisted of two parallel electrodes separated by 2 mm.^[^ ^15^, ^38^ ^]^ To prepare the electrodes, slurry of as‐prepared carbons, PVDF and carbon black with a mass ratio of 8:1:1 in NMP were coated onto a titanium foil with a size of 6 cm × 6 cm, followed by drying at 60 °C for 24 h. The mass of active material in each electrode was ≈40 ± 3 mg. For a typical run, 100 mL of CuCl_2_ solution with initial concentrations of 25–400 ppm for Cu^2+^ was continuously pumped into the ECP cell at a flow rate of 15 mL min^–1^. The voltage between the two electrodes was provided by CHI 760E, which was set at ≈0.4–1.4 V for 420 min followed by reset to 0 V for another 420 min. Prior to the electrochemical removal experiment, the test solution was circulated in the ECP for 12 h without applying voltage to allow physical adsorption. In order to determine the Cu^2+^ concentration during charging, 0.5 mL of the solution was collected at set intervals, diluted by a factor of three, and detected by ICP‐OES (Avio 200). The initial pH of the solution was 5.75 and was adjusted by HCl to explore the effect of pH condition on the removal property of Cu^2+^. The removal capacity (*Q*, mg g^–1^) of Cu^2+^ can be calculated according to Equation (6):

(6) $$Q=\frac{\left(C_{0}-C_{t}\right)\times V_{m}}{m}$$

where *C*_0_ (ppm) is the initial Cu^2+^ concentration of the solution, *C_t_* (ppm) is the Cu^2+^ concentration at time *t*, *V_m_* (L) is the volume of the solution, and *m* (g) is the mass of active material in cathode. In the discharging step, the desorption efficiency (*η*) can be calculated according to Equation (7):

(7) $$\eta =\frac{C_{d}-C_{t}}{C_{0}-C_{t}}$$

where *C_d_* (ppm) is the concentration of Cu^2+^ at the end of discharging step.

To assess the selectivity in the presence of multiple ions, a solution containing CuCl_2_, PbCl_2_, CdCl_2_, and NaCl with the initial concentration of 0.5 mmol L^–1^ for each cation was used. The voltage was set at 0.6–1.2 V for 420 min. The selectivity coefficient (*S*) of Cu^2+^ against the competing ion was calculated via Equation (8):

(8) $$S_{\mathrm{Cu}/M}=\frac{\left(C_{0,\mathrm{Cu}}-C_{t,Cu}\right)/C_{0,Cu}}{\left(C_{0,M}-C_{t,M}\right)/C_{0,M}}$$

where *C*_0,Cu_ and *C*_0,M_ (mmol L^–1^) are the initial concentration of Cu^2+^ and competing ions, respectively. *C_t,_*_Cu_ and *C_t,_*_M_ (mmol L^–1^) are the concentration of Cu^2+^ and competing ions at time *t*, respectively.

The energy consumption (*E*, Wh g^–1^) for the removal of Cu^2+^ was determined by Equation (9):

(9) $$E=\frac{V\times \int_{0}^{t}Idt}{3.6\times \left(C_{0}-C_{t}\right)\times V_{m}}$$

where *V* (V) is the applied voltage, *I* (A) is the current response (A), and *t* (s) is the charging time.

The potentials of cathodes and anodes were measured in an electrochemical cell that mimics the ECP as shown in Figure S15a, Supporting Information: two identical carbonaceous electrodes served as the working and counter electrodes which were separated by 2 mm (the same as in the ECP). An Ag/AgCl electrode was used as the reference electrode. The cell was connected to an electrochemical workstation with two channels (i.e., P1 and P2). A constant voltage (*V*_cell_) was applied to the working and counter electrodes using Channel P1 and the open‐circuit potential of the anode (P^+^) versus the reference electrode was monitored using Channel P2. The potential of the cathode (P^–^) was determined by Equation (10):

(10) $$P^{-}=P^{+}-V_{\mathrm{cell}}$$

### Statistical Analysis

To determine the chemical composition of the materials, the XPS spectra were fitted with XPS PEAK. To calculate the electrochemical capacitance based on the galvanostatic charge–discharge method, the iR drop was first identified from the discharge curve using the raw data provided by the CHI 760E software, through which the discharge time and the corresponding discharge potential were determined and subtracted. The removal capacity of the cations (Na^+^, Cu^2+^, Pb^2+^, and Cd^2+^) were calculated based on the variation of the concentration during the electrochemical deionization tests, which were conducted by collecting 0.5 mL of the solution from the deionization system, diluted by a factor of three and measured by ICP‐OES (Avio 200). All tests were repeated for three times, through which the mean values and standard deviation were determined.

## Conflict of Interest

The authors declare no conflict of interest.

## Supporting information

Supporting Information

## Data Availability

The data that support the findings of this study are available from the corresponding author upon reasonable request.
