# Supplementary material for: Potential‐Mediated Recycling of Copper From Brackish Water by an Electrochemical Copper Pump
Source: Adv Sci (Weinh). 2022 Aug 26;9(30):2203189. doi: 10.1002/advs.202203189 (PMC9596855; doi:10.1002/advs.202203189)
Supplement: Supplementary file 1 — Supporting Information [file ADVS-9-2203189-s001.pdf]

ADVANCED SCIENCE
Open Access

Supporting Information

for *Adv. Sci.*, DOI 10.1002/advs.202203189

Potential-Mediated Recycling of Copper From Brackish Water by an Electrochemical Copper Pump

*Hai Deng, Wenfei Wei, Lei Yao, Zijian Zheng, Bei Li, Amr Abdelkader and Libo Deng**

Support imformation

Potential-Mediated Recycling of Copper from Brackish Water by an Electrochemical Copper Pump

Hai Deng[1], Wenfei Wei[1,2], Lei Yao[2], Zijian Zheng[3], Bei Li[4], Amr Abdelkader[5], and Libo Deng*[1]

[1]College of Chemistry and Environmental Engineering, Shenzhen University, Shenzhen, Guangdong 518060, P. R. China

[2]Shenzhen Key Laboratory of Special Functional Materials, Shenzhen Engineering Laboratory for Advanced Technology of Ceramics, Guangdong Research Center for Interfacial Engineering of Functional Materials, College of Materials Science and Engineering, Shenzhen University, Shenzhen 518060, P. R. China

[3]Institute of Textiles and Clothing, and Research Institute for Smart Energy, The Hong Kong Polytechnic University, Hong Kong SAR, China

[4]College of Biology and the Environment, Nanjing Forestry University, Nanjing 210037, China

[5]Department of Design and Engineering, Faculty of Science & Technology, Bournemouth University, Poole, Dorset BH12 5BB, United Kingdom

* Corresponding author: Denglb@szu.edu.cn; Tel: +86-755-26536157

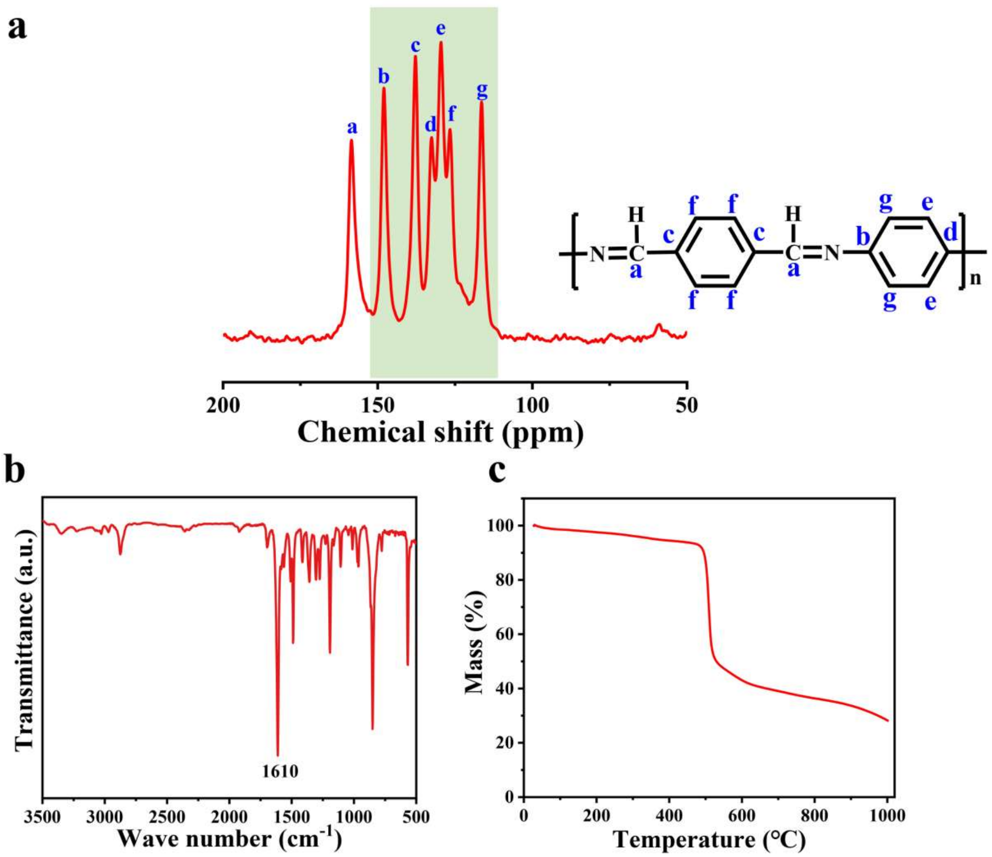

Figure S1. a) ^{13}C-NMR spectrum and b) FTIR spectrum, and c) TGA curve of PPDPA copolymer.

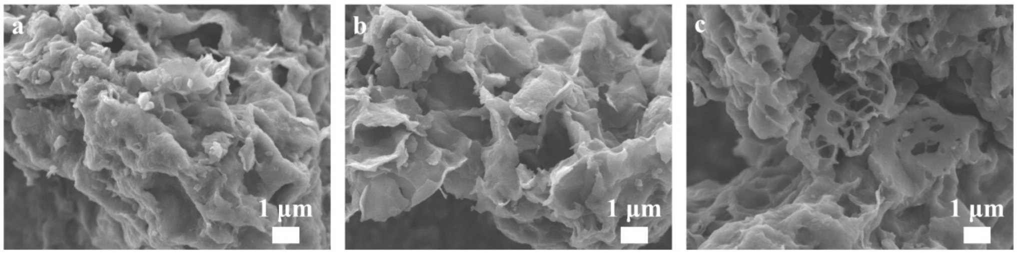

Figure S2. SEM images of: a) NHC, b) KNHC-1, and c) KNHC-2.

Upon KOH activation, the PPDPA-derived carbon evolved from a dense and laminated structure to small flakes (Figure S2), and finally into carbon nanosheets which is the intrinsic morphology of graphitic domain as can be seen from Figure S3a. It is the deep etching of the less stable regions by KOH that exposes the intrinsic nanosheets. TEM inspection revealed that highly crumbled and curled nanosheets consisted of graphitic domains are dispersed in the amorphous matrix (Figure S3b). Elemental mapping images (Figure S3c-e) suggested that heteroatoms N and O, that could improve the conductivity and wettability of the carbonaceous materials, are uniformly distributed in KNHC-3.

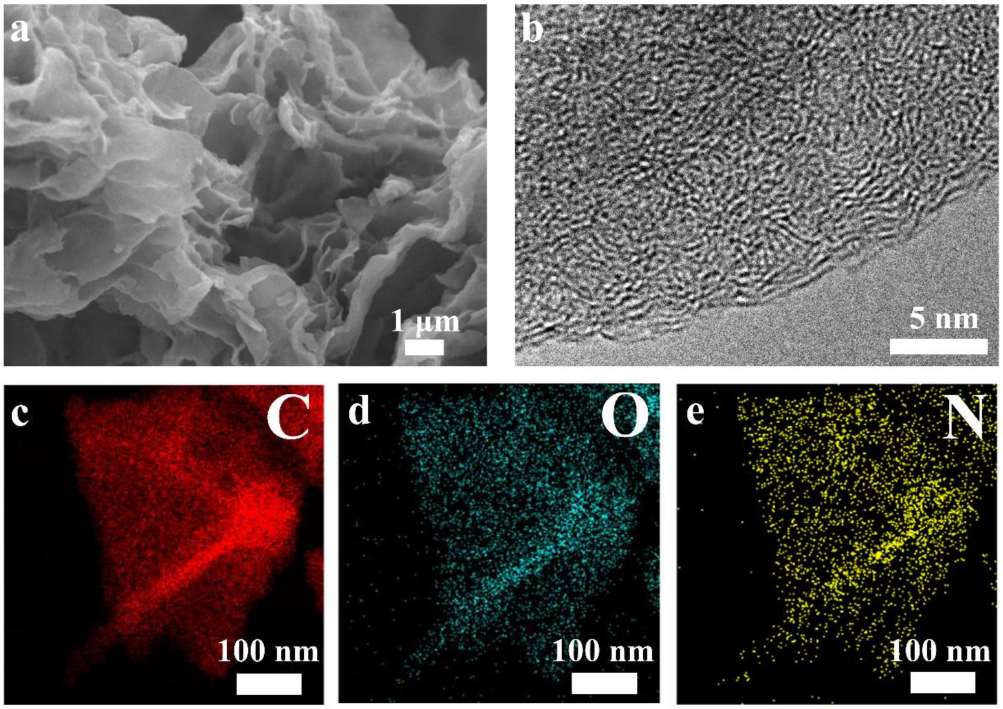

Figure S3. a) SEM image, b) TEM image, and c-e) the corresponding elemental mapping images of KNHC-3.

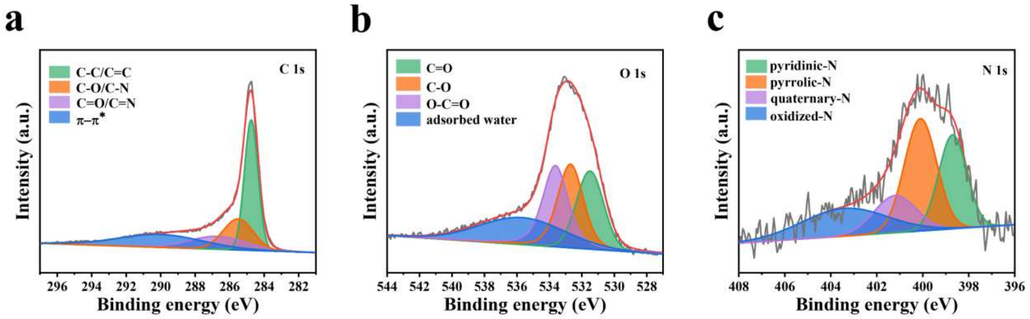

Figure S4. a) C 1s, b) O 1s and c) N 1s spectra of KNHC-3.

The C 1s spectrum could be divided into four individual peaks (Figure S4a), characteristic of C-C/C=C (284.8 eV), C-O/C-N (285.5 eV), C=N/C=O (286.7 eV), and π−π* transition (290.0 eV), respectively. The O 1s spectrum (Figure S4b) could be divided into four peaks, representing C=O (531.5 eV), C–O (532.7 eV), O–C=O (533.7 eV), and absorbed water (535.7 eV), respectively. In the case of the N 1s spectrum (Figure S4c), it could also be divided into four individual peaks, corresponding to pyridinic-N (398.6 eV), pyrrolic-N (400.1 eV), quaternary-N (401.2eV), and oxidized-N (403.1 eV), respectively. It is generally recognized that the presence of nitrogen species in the carbon matrix not only enhances the conductivity and wettability of the materials but also improves the ability for capturing cations.

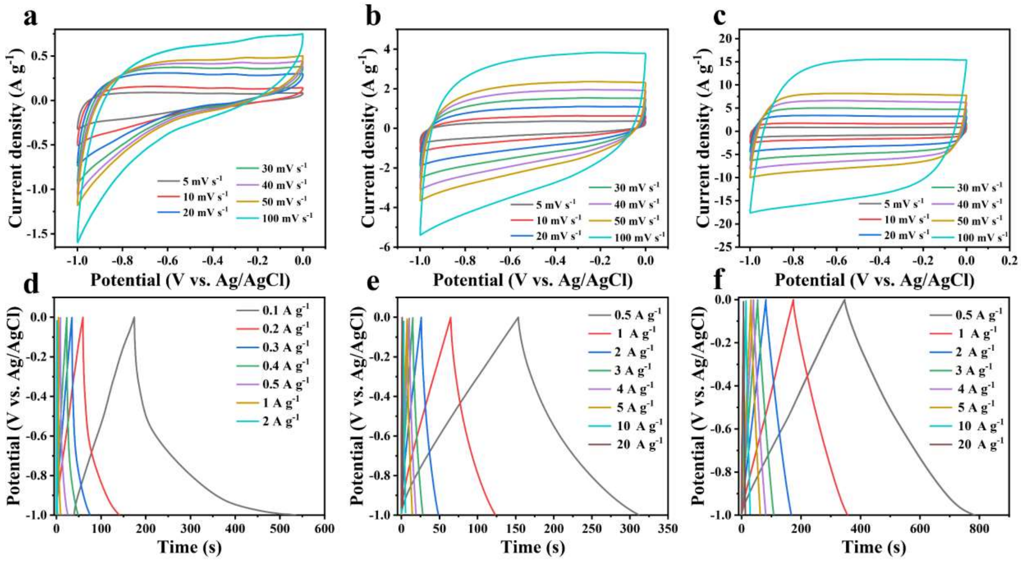

Figure S5. CV curves of: a) NHC, b) KNHC-1, and c) KNHC-2; GCD curves of: d) NHC, e) KNHC-1, and f) KNHC-2.

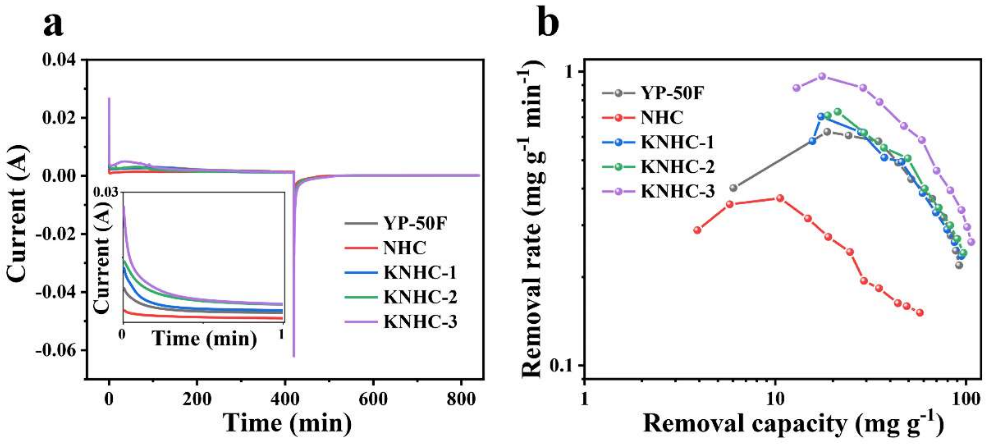

Figure S6. a) The current response and b) Ragone plots for different electrodes.

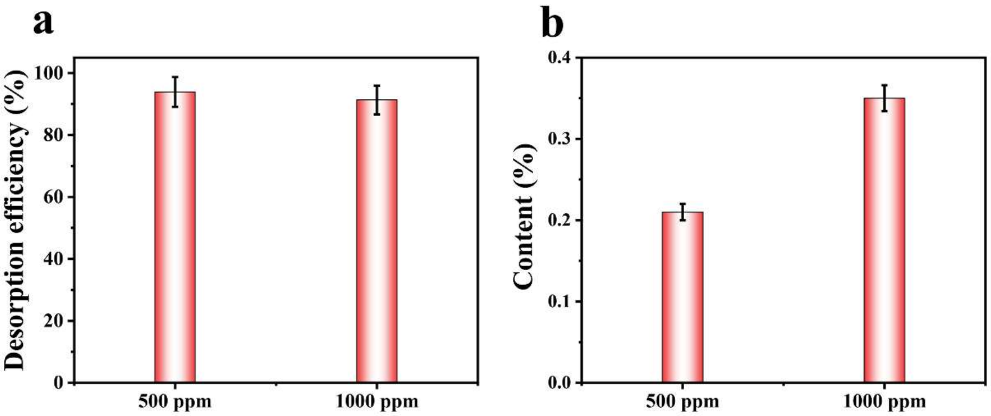

Figure S7. a) Desorption efficiency and b) atomic content of residual Cu on the electrode surface after operating at -1.2 V in 500 ppm and 1000 ppm Cu^{2+} solutions. The error bars represent the standard deviation (sample size: 3).

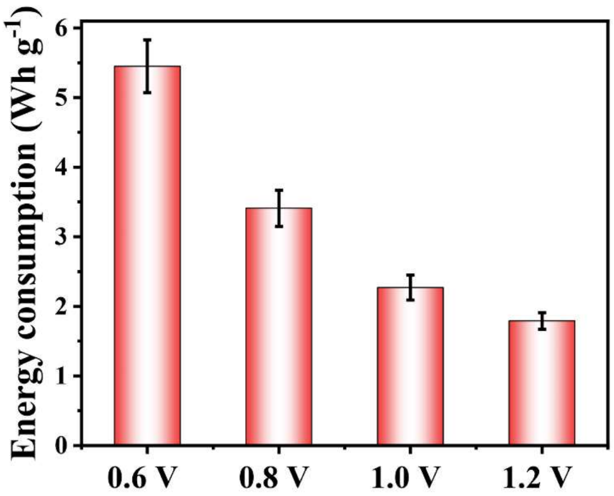

Figure S8. The energy consumption for removal of Cu by the ECP based on KNHC-3 at different voltages. The error bars represent the standard deviation (sample size: 3).

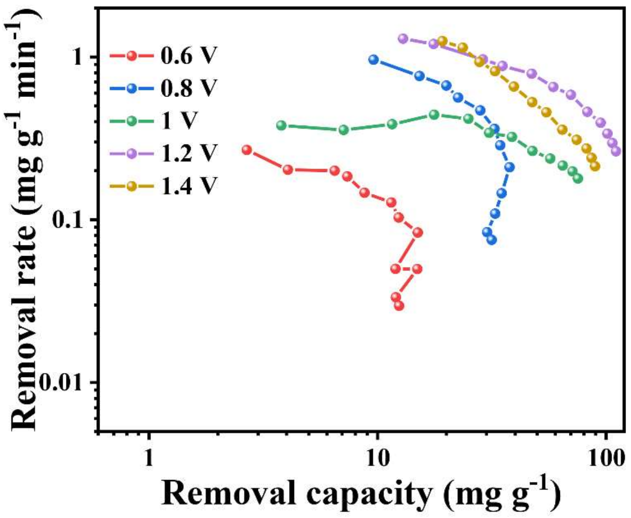

Figure S9. Ragone plots for KNHC-3 electrode at different voltages with an initial Cu^{2+} concentration of 50 ppm.

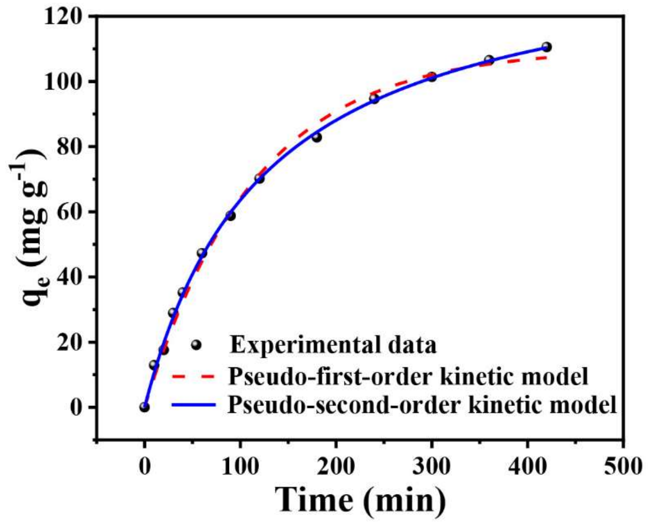

Figure S10. Kinetics isotherms of KNHC-3 electrode at 1.2 V with an initial Cu^{2+} concentration of 50 ppm.

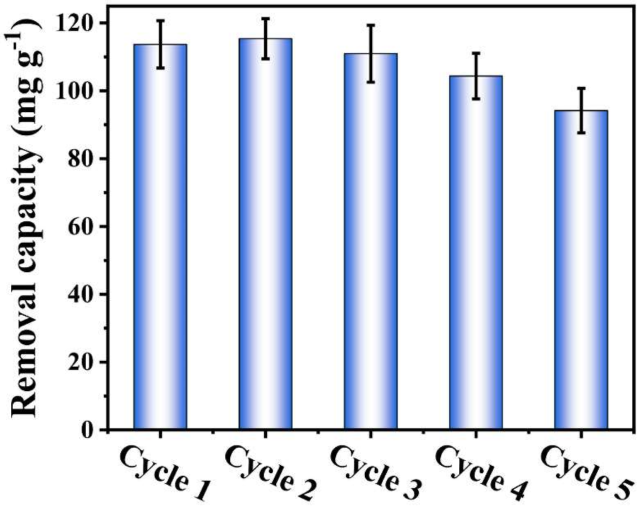

Figure S11 The Cu^{2+} removal capacity of the ECP based on KNHC-3 over the repeated charging-discharging cycles (Charging: 1.2 V, discharging: 0 V, initial concentration: 50 ppm). The error bars represent the standard deviation (sample size: 3).

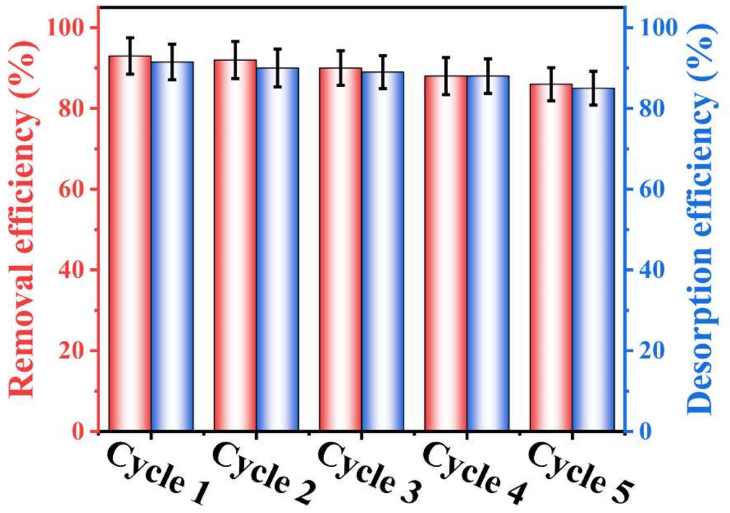

Figure S12. The Cu^{2+} removal and desorption efficiency of the ECP based on KNHC-3 over the repeated charging-discharging cycles (Charging: 1.2 V, discharging: -1.2 V, source solution: 50 ppm, recovery solution: 1000 ppm). The error bars represent the standard deviation (sample size: 3).

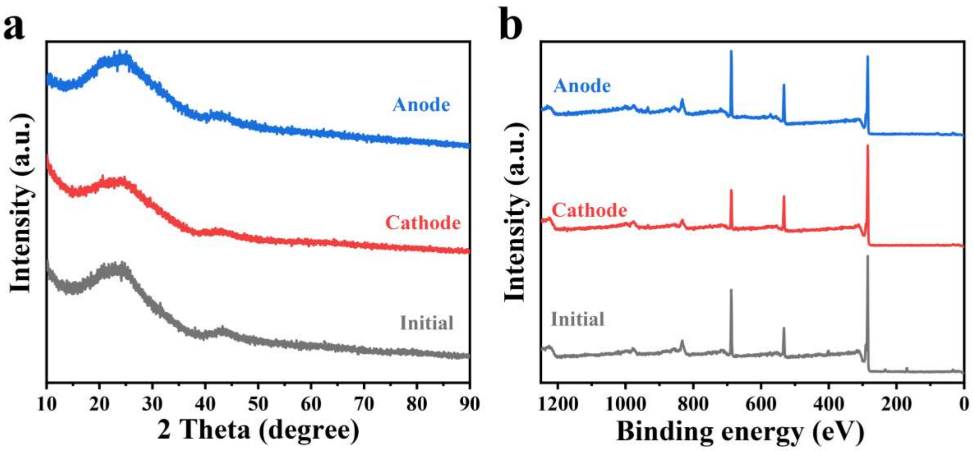

Figure S13. a) XRD patterns and b) XPS spectra after five charging-discharging cycles.

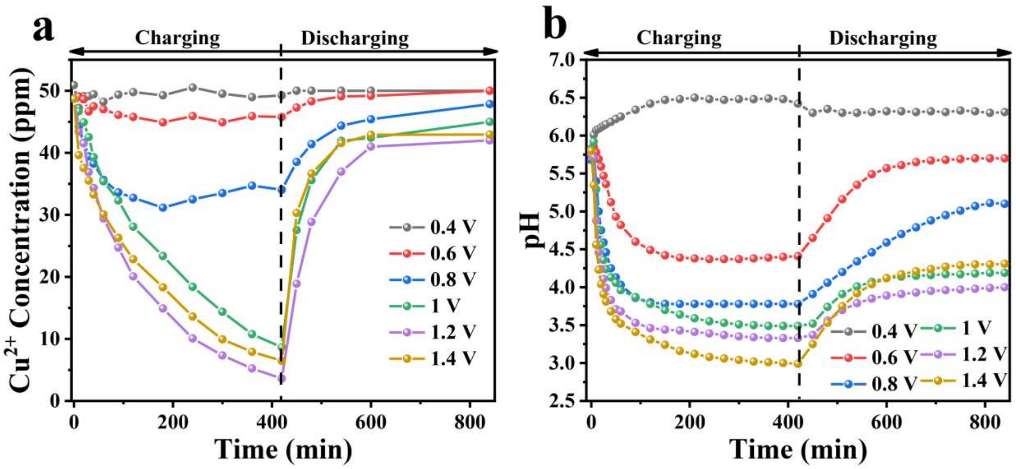

Figure S14. Variation of: a) Cu^{2+} concentration and b) pH during charging and discharging.

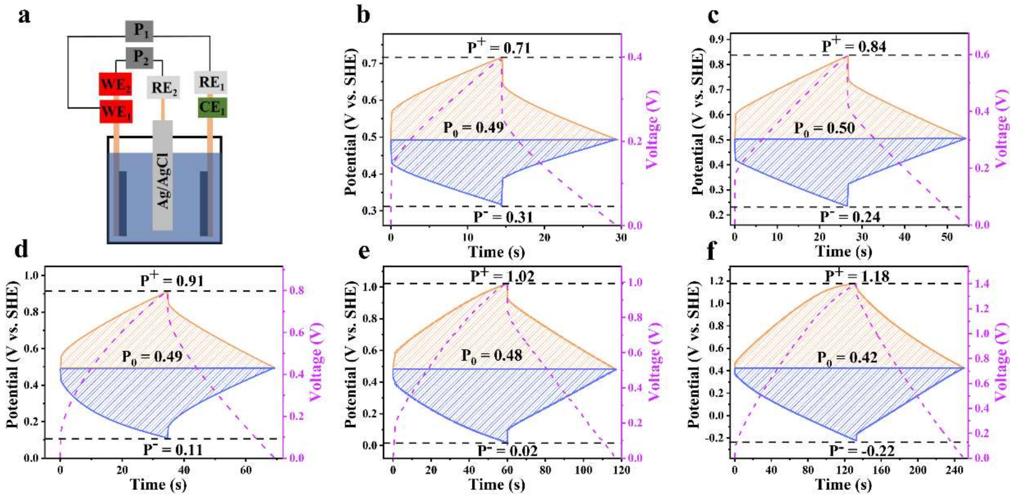

Figure S15. a) Schematic diagrams of the test system, using KNHC-3 as both the working electrode (WE) and counter electrode (CE), Ag/AgCl as the reference electrode (RE), and an electrochemical working station as power (P); GCD curves of anode and cathode in 50 ppm Cu^{2+} solution with supporting electrolyte of Na_2SO_4 at different operational voltages: b) 0.4 V, c) 0.6 V, d) 0.8 V, e) 1 V and f) 1.4 V.

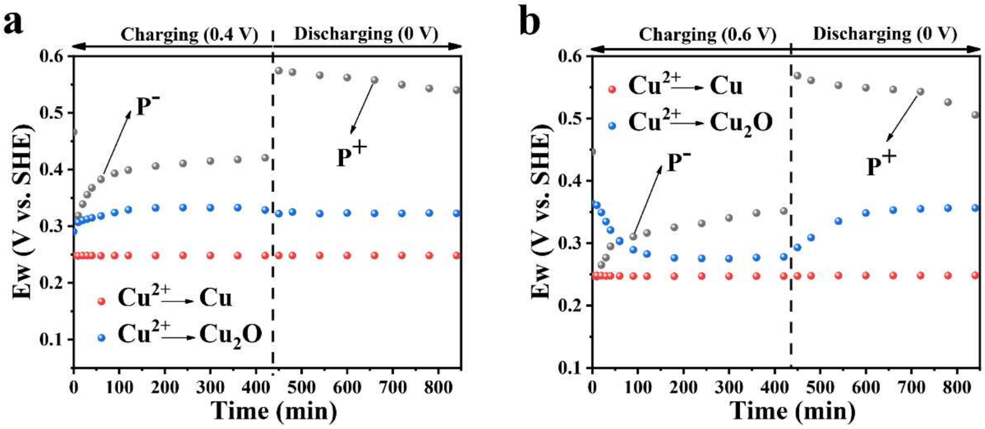

Figure S16. E_w, P^- and P^+ during the deionization tests at: a) 0.4 V and b) 0.6 V.

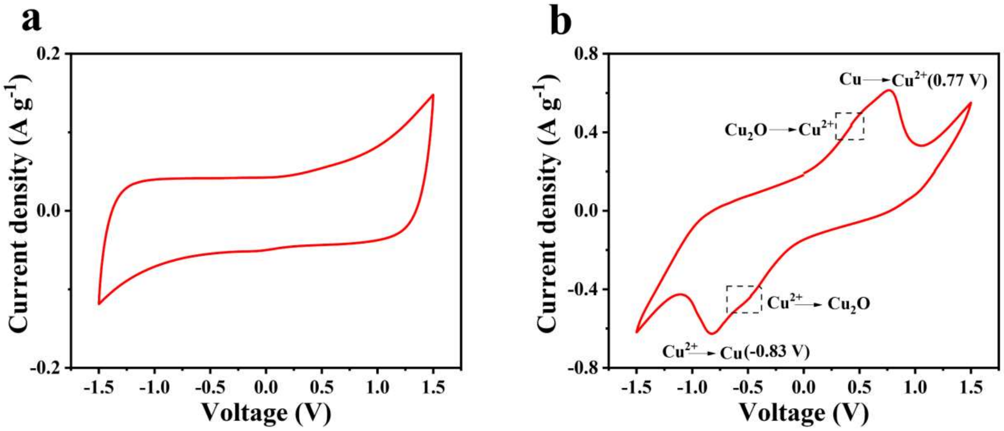

Figure S17. CV curves for the KNHC-3 electrode in CDI system acquired at 2 mV s^{-1} in different solutions: a) 100 mM Na_2SO_4 and b) 50 mM Cu^{2+} with supporting electrolyte of 100 mM Na_2SO_4.

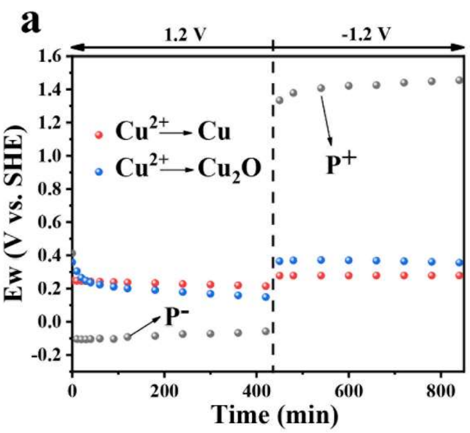

Figure S18. E_w and P^- in 50 ppm Cu^{2+} solutions, and P^+ at a reversal voltage of -1.2 V in concentrated Cu^{2+} solutions: a) 500 ppm and b) 1000 ppm.

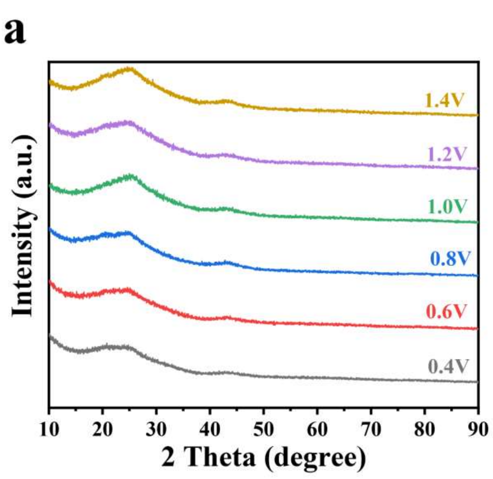

Figure S19. XRD patterns of anodes at different voltages: a) after charging and b) after discharging.

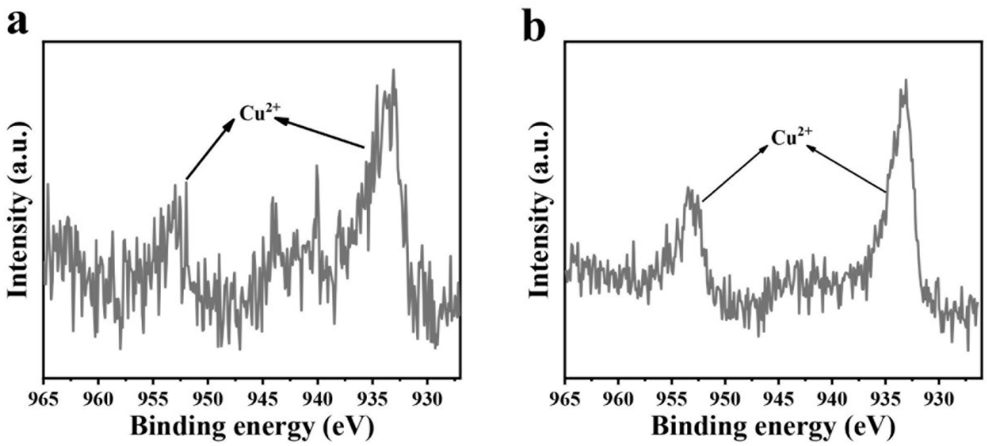

Figure S20. The high-resolution spectrum of Cu 2p of the cathode after charging at: a) 0.4 V and b) 0.6 V.

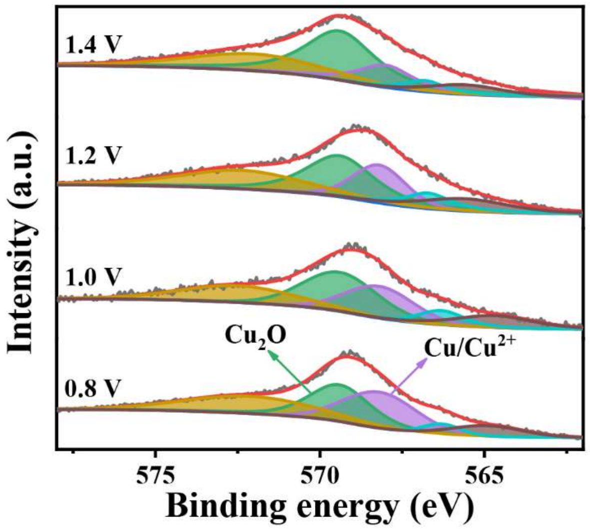

Figure S21. Cu LMM spectra for the cathode charged at different voltages.

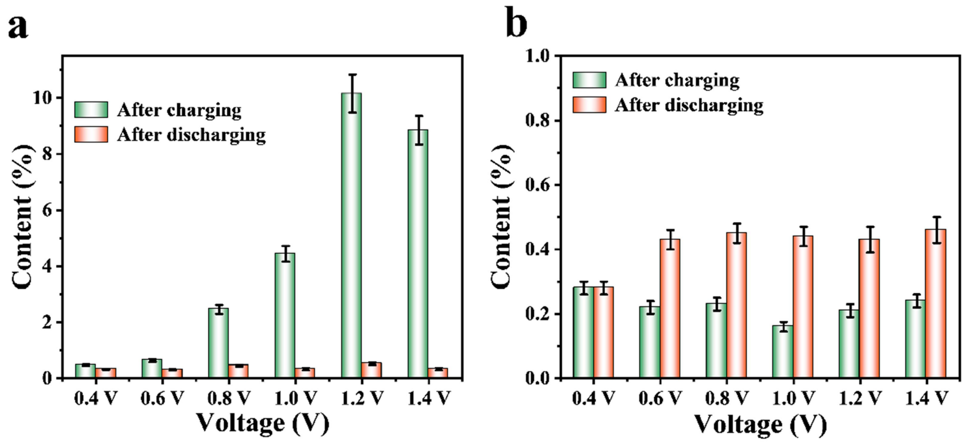

Figure S22. The atomic ratio of Cu element on the electrode surface after charging and discharging: a) cathode and b) anode. The error bars represent the standard deviation (sample size: 3).

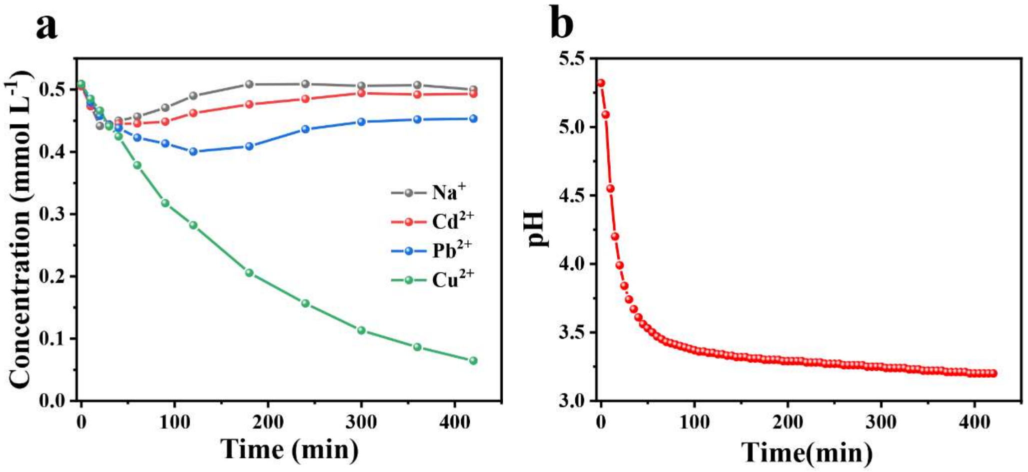

Figure S23. Variation of: a) ion concentration and b) pH during charging at 1.2 V in a mixed solution containing multiple ions.

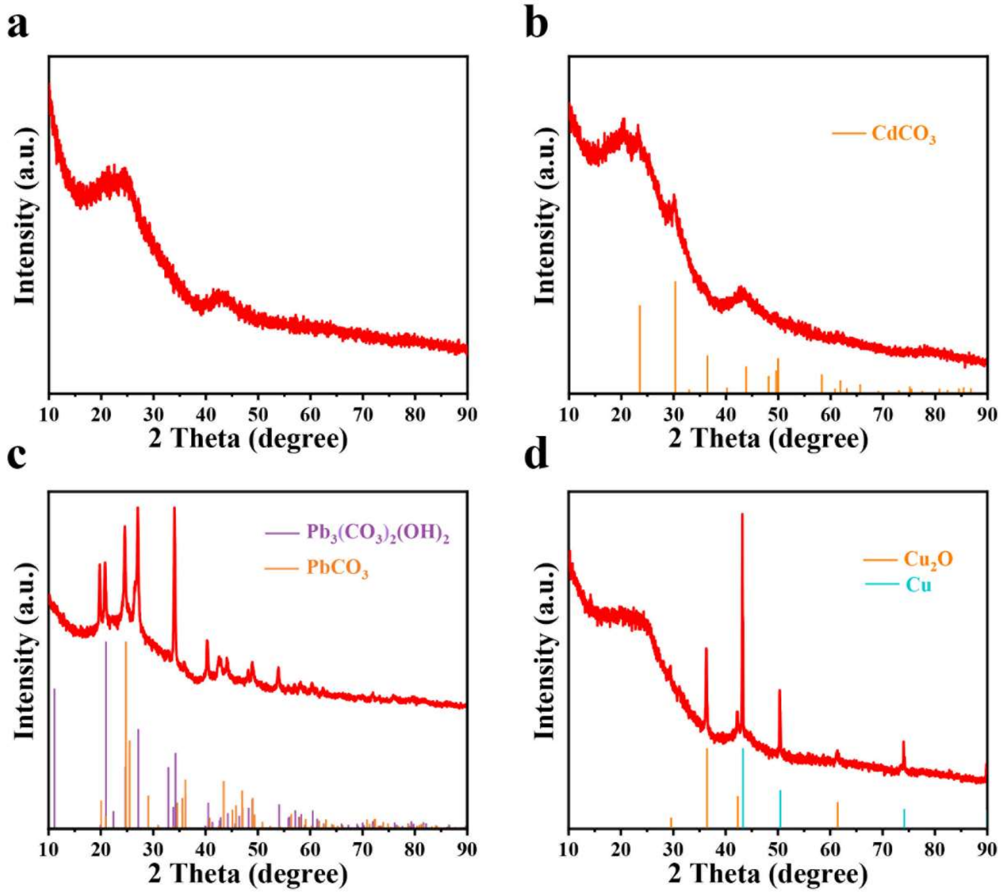

Figure S24. XRD patterns of the cathode after charging at 1.2 V in each individual solution: a) NaCl, b) $CdCl_2$, c) $PbCl_2$ and d) $CuCl_2$.

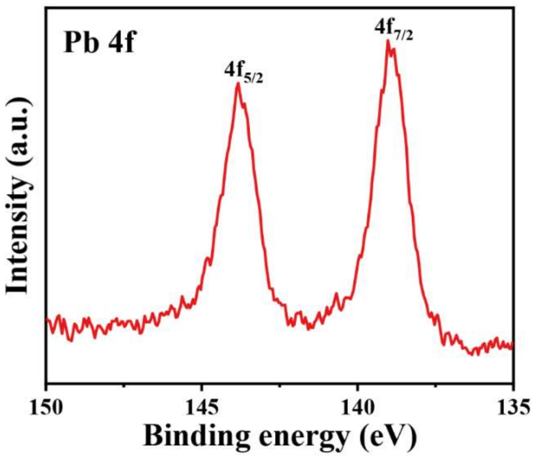

Figure S25. High resolution Pb 4f XPS spectrum of the cathode after charging at 1.2 V in a mixed solution containing multiple ions.

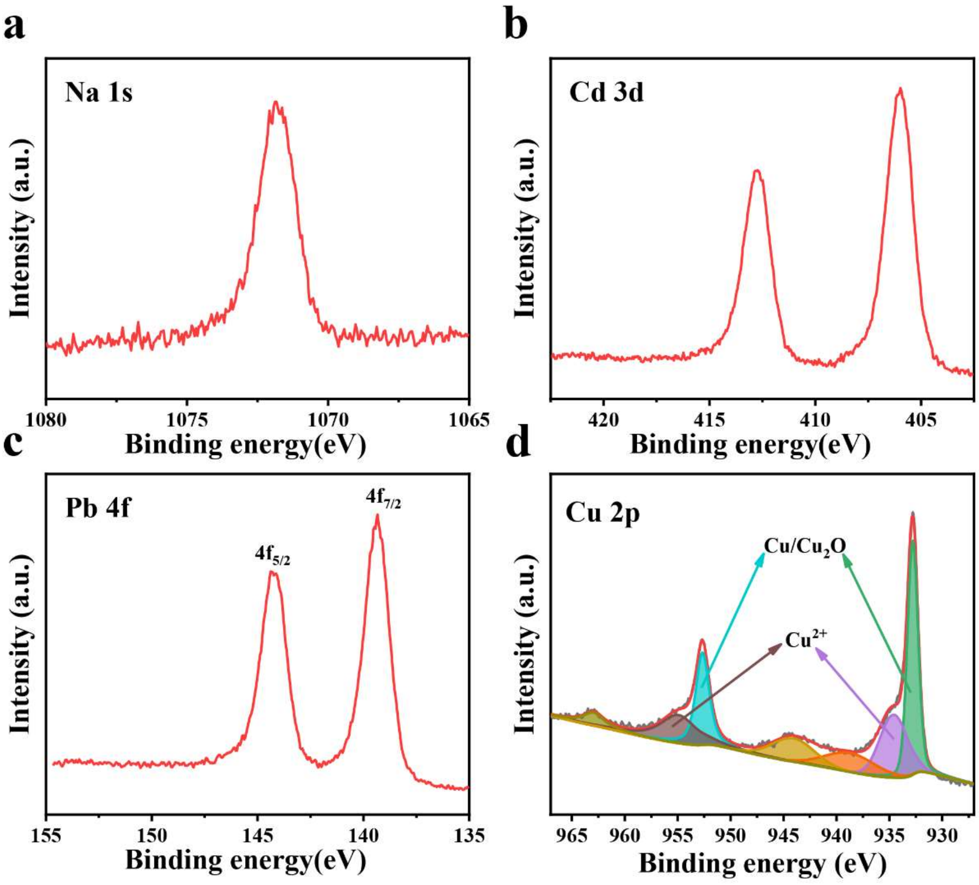

Figure S26. High resolution spectra of cathodes after charging at 1.2 V in each individual solution: a) Na 1s, b) Cd 3d, c) Pb 4f, and d) Cu 2p.

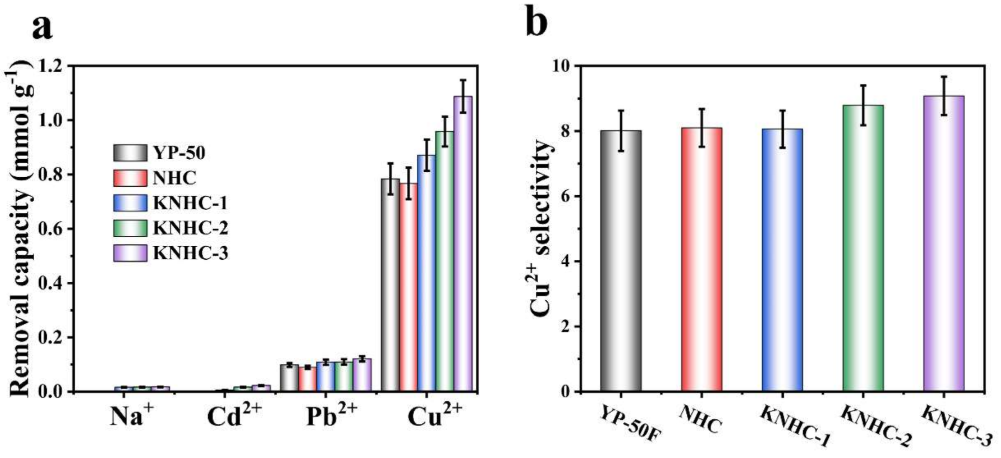

Figure S27. a) The removal capacities of the carbons towards four types of cations and b) the selectivity coefficient of Cu^{2+} versus Pb^{2+} for all carbons tested in mixed solution at 1.2 V. The error bars represent the standard deviation (sample size: 3).

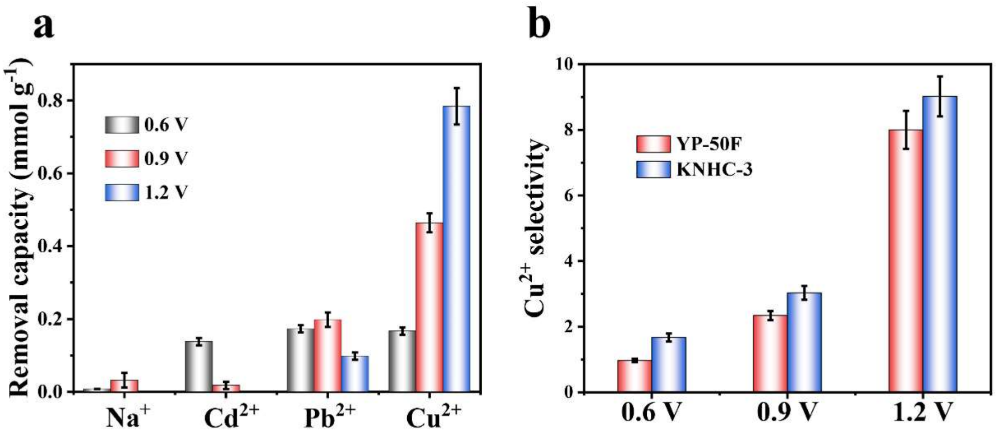

Figure S28. a) The removal capacities towards four types of ions of YP-50F and b) the selectivity coefficient of Cu^{2+} versus Pb^{2+} of YP-50F and KNHC-3 in mixed solution at different voltages. The error bars represent the standard deviation (sample size: 3).

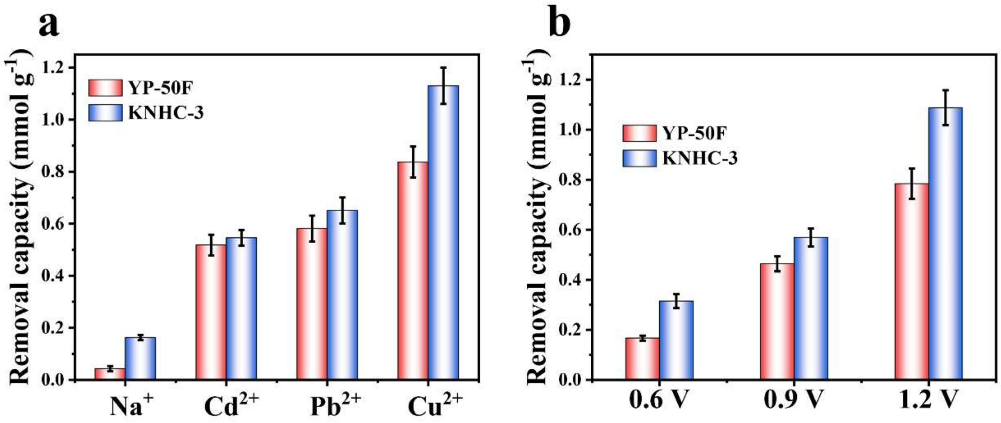

Figure S29. a) The removal capacities towards four types of ions at 1.2 V in each individual solution and b) the Cu^{2+} removal capacities in mixed solution at different voltages of YP-50F and KNHC-3. The error bars represent the standard deviation (sample size: 3).

Table S1. Structural parameters of the as-prepared carbons

Samples	Specific surface are[a)] (m^2 g^{-1})	Pore volume (cm^3 g^{-1}) V_{mirco}[b)]	V_{meso}[c)]	Mean pore diameter (nm)
NHC	534	0.129	0.0957	1.2476
KNHC-1	1590	0.6636	0.4698	1.8862
KNHC-2	2214	0.8366	0.7888	1.9344
KNHC-3	2786	1.1403	1.3984	2.1995

Note: a) Specific surface area and Mean pore diameter were calculated using BET method, b) Micropore volume (V_{mirco}) determined by HK model and c) Mesopore volume (V_{meso}) determined by BJH model.

Table S2. Comparison of the removal capacity of carbon electrode towards Cu^{2+} with the literature data.

Materials	Initial concentration (ppm)	Applied voltage (V)	SAC (mg g^{-1})	Ref.
Activated carbon	50	1.2	2.5	[1]
3-dimensional reduced graphene oxide	50	1	18.1	[2]
Cation-exchange resin derived active carbon	50	1.2	77.8	[3]
N, S-co-doped carbon material	50	1.5	51.0	[4]
N, F-co-doped 3-dimensional reduced graphene oxide	100	1.2	52.4	[5]
Carbon aerogel	100	1.2	29.7	[6]
Polypyrrole/chitosan/carbon nanotube composite	100	0.8	16.8	[7]
Graphene porous carbon nanosheets	100	1.2	15	[8]
Polypyrrole/Graphene	100	1.2	41.51	[9]

Oxide Composite				
Ordered mesoporous carbon	200	0.9	56.62	[10]
KNHC-3	50	1.2	113.7	This work

Table S3. Parameters for pseudo-first-order and pseudo-second-order models.

Sample	Pseudo-first-order		Pseudo-second-order	
KNHC-3	k_1(min^{-1})	0.00872	k_2(g mg min^{-1})	5.573×10^{-5}
	q_e(mg g^{-1})	110.12	q_e(mg g^{-1})	143.26
	R^2	0.996	R^2	0.999

Supplementary References

[1] S. Y. Huang, C. S. Fan, C. H. Hou, *J. Hazard. Mater.* **2014**, *278*, 8.

[2] S. M. You, C. K. Tasi, P. Millet, R. A. Doong, *Sep. Purif. Technol.* **2020**, *251*, 117368.

[3] S. J. Wu, P. J. Yan, W. Yang, J. Zhou, H. Wang, L. Che, P. F. Zhu, *Chemosphere* **2021**, *264*, 128557.

[4] Y. N. Chang, Q. D. Dang, I. Samo, Y. P. Li, X. J. Li, G. X. Zhang, Z. Chang, *RSC Adv.* **2020**, *10*, 4064.

[5] G. S. S. Mamaril, M. D. G. de Luna, K. Bindumadhavan, D. C. Ong, J. A. I. Pimentel, R. A. Doong, *Sep. Purif. Technol.* **2021**, *272*, 117559.

[6] Z. L. Cao, C. Zhang, Z. X. Yang, Q. Qin, Z. H. Zhang, X. D. Wang, J. Shen, *Materials* **2019**, *12*, 1864.

[7] Y. J. Zhang, J. Q. Xue, F. Li, J. Z. Dai, X. Z. Y. Zhang, *Chem Eng Process* **2019**, *139*, 121.

[8] H. Wang, T. T. Yan, J. J. Shen, J. P. Zhang, L. Y. Shi, D. S. Zhang, *Environ. Sci. Nano* **2020**, *7*, 317.

[9] J. Q. Xue, Q. X. Sun, Y. J. Zhang, W. B. Mao, F. G. Li, C. X. Yin, *ACS Omega* **2020**, *5*, 10995.

[10] C.-C. Huang, J.-C. He, *Chem. Eng. J.* **2013**, *221*, 469.
